# Revisiting the two rhythm generators for respiration in lampreys

**DOI:** 10.3389/fnana.2023.1270535

**Published:** 2024-01-05

**Authors:** Kianoush Missaghi, Jean-Patrick Le Gal, Julien Mercier, Martin Grover, Philippe-Antoine Beauséjour, Shannon Chartré, Omima Messihad, François Auclair, Réjean Dubuc

**Affiliations:** ^1^Département de Neurosciences, Université de Montréal, Montréal, QC, Canada; ^2^Groupe de Recherche en Activité Physique Adaptée (GRAPA), Département des Sciences de l’Activité Physique, Université du Québec à Montréal, Montréal, QC, Canada

**Keywords:** respiration, respiratory generator, neuroanatomy, electrophysiology, brainstem, DAMGO, substance P, lamprey

## Abstract

In lampreys, respiration consists of a fast and a slow rhythm. This study was aimed at characterizing both anatomically and physiologically the brainstem regions involved in generating the two rhythms. The fast rhythm generator has been located by us and others in the rostral hindbrain, rostro-lateral to the trigeminal motor nucleus. More recently, this was challenged by researchers reporting that the fast rhythm generator was located more rostrally and dorsomedially, in a region corresponding to the mesencephalic locomotor region. These contradictory observations made us re-examine the location of the fast rhythm generator using anatomical lesions and physiological recordings. We now confirm that the fast respiratory rhythm generator is in the rostro-lateral hindbrain as originally described. The slow rhythm generator has received less attention. Previous studies suggested that it was composed of bilateral, interconnected rhythm generating regions located in the caudal hindbrain, with ascending projections to the fast rhythm generator. We used anatomical and physiological approaches to locate neurons that could be part of this slow rhythm generator. Combinations of unilateral injections of anatomical tracers, one in the fast rhythm generator area and another in the lateral tegmentum of the caudal hindbrain, were performed to label candidate neurons on the non-injected side of the lateral tegmentum. We found a population of neurons extending from the facial to the caudal vagal motor nuclei, with no clear clustering in the cell distribution. We examined the effects of stimulating different portions of the labeled population on the respiratory activity. The rostro-caudal extent of the population was arbitrarily divided in three portions that were each stimulated electrically or chemically. Stimulation of either of the three sites triggered bursts of discharge characteristic of the slow rhythm, whereas inactivating any of them stopped the slow rhythm. Substance P injected locally in the lateral tegmentum accelerated the slow respiratory rhythm in a caudal hindbrain preparation. Our results show that the fast respiratory rhythm generator consists mostly of a population of neurons rostro-lateral to the trigeminal motor nucleus, whereas the slow rhythm generator is distributed in the lateral tegmentum of the caudal hindbrain.

## 1 Introduction

Respiration is a vital rhythmic motor activity that results from complex interactions between different respiratory centers in the brainstem (Dutschmann and Dick, 2012; Feldman et al., 2013; Smith et al., 2013; Richter and Smith, 2014; Feldman and Kam, 2015; Ramirez et al., 2016; Del Negro et al., 2018). In mammals, the respiratory centers are distributed as a lateral column extending rostro-caudally throughout the hindbrain to the rostral spinal cord with a rhythmogenic core located in the medulla oblongata (Feldman and Del Negro, 2006; Alheid and McCrimmon, 2008; Smith et al., 2009; Dutschmann and Dick, 2012). The rhythm-generating interneurons are primarily found in the pre-Bötzinger complex (inspiration) (Smith et al., 1991; Feldman et al., 2013), but also in the conditionally active retrotrapezoid nucleus and the parafacial respiratory group (active expiration) (Pagliardini et al., 2011; Huckstepp et al., 2018).

The evolutionary origin of the respiratory generators of mammals is still unclear. Studies performed in fishes and amphibians revealed that the respiratory generators are located in the caudal hindbrain, a region homologous to the medulla oblongata of mammals (Duchcherer et al., 2010; Baghdadwala et al., 2015). In the lamprey, an aquatic basal vertebrate, gill ventilation is also controlled by populations of neurons in the hindbrain. Two respiratory rhythms are observed: a fast and a slow rhythm (Russell, 1986; Martel et al., 2007; Gariépy et al., 2012a). The fast respiratory rhythm (around 1 Hz) is thought to be the functional equivalent of eupnea in mammals (Rovainen, 1985; Russell, 1986; Missaghi et al., 2016). Both in man and in most other mammals, spontaneous breathing is also periodically interrupted by “deep breaths” or “sighs” (Cherniack et al., 1981; see also Haldane et al., 1919; Larrabee and Knowlton, 1946; Reynolds, 1962; Reynolds and Hilgeson, 1965; Bartlett, 1971; Glogowska et al., 1972). Therefore, the presence of a slow rhythm in addition to a fast respiratory rhythm is not unique to lampreys. Based on electrophysiological data and lesion experiments, a number of studies spanning over four decades have located the fast rhythm generator of lampreys in the region lateral to the trigeminal motor nucleus, from the rhombencephalic isthmus near the large reticulospinal isthmic cell 2 (cell I2) to the caudal level of exit of the vestibular nerve, with some evidence that it is distributed over this whole lateral region of the rostral hindbrain (Homma, 1975; Kawasaki, 1979, 1984; Rovainen, 1983, 1985; Thompson, 1985; Russell, 1986; Martel et al., 2007; Mutolo et al., 2007, 2010; Gariépy et al., 2012a,b). The fast rhythm generator has been shown to be sensitive to hypoxia through unknown mechanisms (Rovainen, 1996), but remains unaffected by hypercapnia (Hoffman et al., 2016). Because of its location rostro-lateral to the trigeminal motor nucleus, the fast rhythm generator was renamed the paratrigeminal respiratory group (pTRG; Mutolo et al., 2007). In the latter article, respiration-related activity was recorded between cell I2 and the anterior octavomotor nucleus. It was proposed more recently (Cinelli et al., 2013) that the fast rhythm generator was located in a more medial and rostral position, in an area dorsal to the large reticulospinal isthmic cell 1 (cell I1), a region previously described as being part of the mesencephalic locomotor region of lampreys (MLR, Le Ray et al., 2003). It is noteworthy that the isthmic region of the lamprey is small and anatomically complex, and because the roof of the isthmus is sometimes cut open to gain better access to cell populations, it adds to the complexity and confusion in locating different structures in the area. Because our study was aimed at better defining the slow rhythm generator and because the fast and slow rhythm generators display important interactions (Martel et al., 2007), we felt that the first step in the present study should focus on carefully re-examining the location of the fast rhythm generator.

The slow respiratory rhythm is characterized by stronger contractions of the gills that result from long bursts of discharge occurring less frequently in the respiratory motoneurons (Martel et al., 2007). This activity occurs as episodes of single or multiple successive bursts. Other than its direct contribution to gill ventilation, the slow rhythm has been proposed to play a role in cleaning the branchial apparatus from particles interfering with gas exchanges (Rovainen, 1977, 1985). In larval lampreys, the slow rhythm generator is sensitive to CO_2_/pH and it is thought that this characteristic could play a role in preventing the accumulation of metabolically-produced CO_2_ in these animals that live in narrow tube burrows dug in soft sediments at the bottom of streams (Hoffman et al., 2016). The authors also proposed that this CO_2_-sensitive generator could constitute an ancestral precursor for air-breathing in vertebrates. Several lesion studies have localized the slow rhythm generator in the caudal hindbrain (Kawasaki, 1979, 1984; Thompson, 1985; Martel et al., 2007), which is homologous to the mammalian medulla oblongata (Brocard and Dubuc, 2003; Butler and Hodos, 2005), a structure that contains the respiratory rhythm-generating regions. Lesion studies have also revealed that lampreys have one slow rhythm generator on each side of the caudal hindbrain and that the two are synchronized, suggesting that they are interconnected. The slow rhythm burst episodes also momentarily stop the fast rhythm, which further suggests ascending projections from the caudal hindbrain to the pTRG in the rostral hindbrain (Martel et al., 2007). The medial reticular formation is not necessary for the respiratory rhythm generation, the lateral tegmentum being sufficient (Rovainen, 1985). Despite the information already gathered on the slow respiratory rhythm generator in the caudal hindbrain, a lot remains to be clarified.

In this study, we first used anatomical, physiological and lesion experiments to identify the region in the rostral hindbrain that is responsible and necessary for generating the fast rhythm. The data presented here show that the fast rhythm generator is not located dorsal to cell I1, but is rather located closer to cell I2, in an area rostro-lateral to the trigeminal motor nucleus. We also used the same techniques to better characterize the region of the caudal hindbrain that contains the slow rhythm generator. We found longitudinally distributed populations of neurons that could account for the synchrony between the two sides and for the effects of the slow rhythm generator on the fast rhythm generator. The activation of portions of the lateral tegmentum in the caudal hindbrain triggered bursts of discharge characteristic of the slow rhythm. Inactivation of the same regions stopped the slow rhythm. Responses to glutamate and neuropeptides were also examined. Revisiting the organization of the two rhythm generators for respiration in lampreys is timely as it is now known that two rhythms with differential frequencies are also generated by the respiratory centers in mammals (Tryba et al., 2008). Our results are discussed in relation to what is known of the respiratory generators in mammals.

## 2 Materials and methods

Anatomical and physiological experiments were performed on juvenile (*n* = 82) and adult (*n* = 31) *Petromyzon marinus* (based on Clemens, 2019 for terminology of life stages). The juvenile lampreys were purchased from Acme Lamprey Co. (Harrison, ME), and the adults were collected from the Great Chazy River (NY, USA). The animals were kept at 4–6°C in aerated tap water treated to remove chlorine and heavy metals. All surgical and experimental procedures conformed to guidelines of the Canadian Council on Animal Care and were approved by the Animal Care and Use Committee of the Université de Montréal and the Université du Québec à Montréal. Care was taken to minimize the number of animals used and their suffering.

The animals were deeply anesthetized with tricaine methanesulfonate (MS-222, Sigma, E10521, 100 mg/l in fresh water) and their brainstem was dissected out by complete transverse sections just above the mesencephalon and below the obex, with the underlying cranium kept for support. The resulting dissection is referred to as the *in vitro* isolated brainstem preparation. During the dissection and the experiments, the preparations were kept at 7–9°C in continuously renewed oxygenated Ringer’s solution of the following composition (in mM): NaCl, 130; KCl, 2.1; CaCl_2_, 2.6; MgCl_2_, 1.8; HEPES, 4; dextrose, 4; NaHCO_3_, 1. The pH was adjusted to 7.4.

### 2.1 Lesion experiments

Lesions of the brainstem were carried out to determine whether the area around cell I1 was the region generating the fast respiratory rhythm. In this case, the *in vitro* isolated brainstem preparation described above was modified. A mid-sagittal section through the roof of the isthmus was first performed to visualize cell I1. Micro-scissors were then used to cut out the mesencephalon and the middle portion of the rostral hindbrain comprising cell I1. On some preparations, the whole gill apparatus was kept for visual monitoring of the actual breathing movements of the gills before and after the lesions.

### 2.2 Electrophysiological experiments

Extracellular recordings of respiratory motoneurons were carried out using glass electrodes filled with Ringer’s and placed over the respiratory motoneuron nuclei, which are readily accessible on the dorsal surface of the lateral tegmentum (40–80 μm tip diameter in juvenile lampreys; 125 μm in adult lampreys). The electrodes were connected to an AC amplifier model 1800 (A-M Systems; Sequim, WA; low cut-off: 100 Hz; high cut-off: 1 kHz). Respiratory motoneurons were also recorded intracellularly with sharp glass microelectrodes (4 M KAc; 80–130 MΩ) and the signals were amplified with an Axoclamp 2A amplifier (Axon Instruments, Foster City, CA; sampling rate: 10 kHz).

To investigate the presence of the fast respiratory rhythm in the rostral hindbrain, glass electrodes filled with Ringer’s (20–40 μm tip diameter) were used in 5 preparations. The area around cell I1 was probed by applying the tip of the electrode on the ependymal surface all around the giant cell. The neurons that have been proposed by Cinelli et al. (2013) to belong to the fast rhythm generator in the area are located just beneath the ependymal layer dorsal to cell I1, near the sulcus limitans. Any activity of these cells should be picked up by the method used here, the same way the activity of respiratory motoneurons is. The area closer to cell I2 was also probed by gradually lowering the electrode through the sulcus limitans, underneath the alar plate at a rather steep angle toward more ventral and lateral locations. From the ependymal surface to the deeper locations, each descent of the recording electrode proceeded by small steps at each of which the recorded signal was assessed for rhythmic respiratory activity. The initial point of entry in the sulcus limitans was moved gradually along the whole lateral edge of the trigeminal motor nucleus.

In some adult lampreys (*n* = 5), the site for the fast respiratory rhythm generator was satisfactorily located and labeled. This site was defined as the location of optimal signal of respiratory bursts synchronized with respiration recorded from the IX motor nucleus. The recording extracellular electrode was then quickly removed and replaced with a micropipette of the same size filled with Texas Red-dextran amines (Invitrogen, Carlsbad, CA, D1863, 10,000 MW). The recording electrode left a visible hole in the tissue at the point of entrance in the sulcus limitans which helped lowering the injection micropipette at the same spot from which the optimal signal was recorded. The distance that the tip of the recording electrode and the injection micropipette traveled in the tissue from the point of entrance to the spot of optimal signal was carefully reproduced using the graduations on the micromanipulator. A single injection of tracer was then applied with a Picospritzer (General Valve, Fairfield, NJ) to mark the spot. After a waiting period of 1 min, the micropipette was removed and the preparation was immediately immersed in 4% paraformaldehyde in phosphate buffered saline (PBS: 0.1 M, pH 7.4 with 0.9% NaCl) for 24 h at 4°C and transferred to 20% sucrose in phosphate buffer (0.1 M, pH 7.4) overnight for cryoprotection. The tissue was then cut transversally with a cryostat (25 μm thickness). The sections were collected on ColorFrost Plus slides (Fisher Scientific, Ottawa, ON) and left to dry on a warming plate at 37°C overnight. The next day, the sections were rinsed in PBS 3 times 10 min each, and then incubated in the fluorescent Nissl stain, Neurotrace Green (diluted 1:150 in PBS, N21480, Invitrogen), for 30 min at room temperature for counter-coloration. The sections were then rinsed 3 times in PBS and mounted with Vectashield (H-1000, Vector Laboratories, Newark, CA).

To further characterize the role of the rostro-lateral hindbrain in the generation of the fast respiratory rhythm, the sodium channel blocker, Xylocaine (lidocaine 2%, DIN 02302438, AstraZeneca, Mississauga, ON, Canada) was bilaterally injected in the area dorsal to cell I1, or alternatively in the area around cell I2, in the same animals (*n* = 6). The bilateral injections in either of the two areas were performed sequentially with the same micropipette moved from one side to the other in a matter of seconds.

Other physiological experiments were carried out to determine whether the anatomically identified neurons in the lateral tegmentum of the caudal hindbrain (see next section) played a role in generating the slow rhythm. An isolated *in vitro* caudal hindbrain preparation was used, which included the facial (VII), glossopharyngeal (IX) and vagal (X) motor nuclei, separated from the spinal cord and the rostral hindbrain by complete transections. In this preparation, the respiratory motoneurons recorded extracellularly did not show any respiratory activity related to the fast rhythm generator, but were still strongly activated by the slow rhythm generator.

The lateral tegmentum of the caudal hindbrain preparation was stimulated electrically or chemically while respiratory motoneurons were recorded on the opposite side. Three stimulation sites equidistant from one another were arbitrarily selected to be stimulated along the rostro-caudal axis of the lateral tegmentum: the rostral (VII and IX level), the middle (rostral X level), and the caudal (caudal X level) sites. Homemade glass-coated tungsten microelectrodes (0.8–2 MΩ) were used to deliver trains of stimuli (3 pulses of 5–11 μA; at 30 Hz; every 50–80 s), whereas chemical stimulation was performed by pressure-injecting a glutamatergic agonist, D,L-glutamate (Sigma, G1126, 2.5 mM) (Gariépy et al., 2012a,b) diluted in Ringer’s solution through a glass micropipette using a Picospritzer. The pressure pulses were triggered by a GRASS stimulator S88 (GRASS instruments, Warwick, RI) connected to the Picospritzer. D,L-glutamate was injected using stimulation trains of 5–10 ms pulses delivered at 5 Hz for 5–10 s with a pressure varying between 1 to 4 psi. The trains were repeated every 80–120 s. In other experiments, bilateral injections of Xylocaine (Gariépy et al., 2012a), or of the synthetic opioid peptide with a high μ-opioid receptor specificity, DAMGO, (1 mM, [D-Ala^2^, N-MePhe^4^, Gly-ol]-enkephalin, E 7384, Sigma, Oakville, ON) (Mutolo et al., 2007), or of substance P (1 μM, S 6883, Sigma, Oakville, ON), a neuropeptide of the tachykinin family and agonist of the NK1 receptors, were also performed with the Picospritzer (5–30 ms pulses at 1–4 psi) to modulate the slow rhythm. These bilateral injections were carried out sequentially with the same micropipette moved within a few seconds from one site to the other. Injections of Xylocaine in the rostral and caudal sites were also carried out in the whole-brainstem preparation (see above). In all cases, Fast Green (Fisher Scientific, F-99) was added to the drug solutions to visualize the injection sites as routinely done in other studies (Brocard and Dubuc, 2003; Derjean et al., 2010; Mutolo et al., 2010; Gariépy et al., 2012a,b; Cinelli et al., 2016; Juvin et al., 2016; Ryczko et al., 2017). The volume injected was estimated by measuring the diameter of a droplet ejected in air from the tip of the pipette (tip diameter 10–20 μm) after applying a single pressure pulse (3–4 psi). The volume of the droplet was then calculated by using the equation of a sphere (Le Ray et al., 2003; Brocard et al., 2005; Ryczko et al., 2013). The size of the injections was further controlled visually under a stereomicroscope by carefully monitoring the spread of Fast Green at the level of the injection site in the brain tissue.

In other experiments, substance P (1 μM) (S 6883, Sigma, Oakville, ON) or a cocktail of glutamate receptor antagonists, 6-cyano-7-nitroquinoxaline-2,3-dione (CNQX, Tocris 1045, 30 μM) and (2R)-amino-5-phosphonovaleric acid (AP5, Tocris 3693, 200 μM) were applied to the bath containing the caudal hindbrain preparation. The concentration used was based on previous studies carried out in lampreys (Martel et al., 2007; Mutolo et al., 2007, 2010).

### 2.3 Anatomical experiments

A first series of anatomical experiments was designed to confirm the presence and the location of rostral hindbrain neurons that project to the respiratory motoneurons (*n* = 7). The rostral vagal motoneuron nucleus was first lesioned with a pulled glass micropipette at depth ranging from the cell bodies on the dorsal surface to the distal dendrites in the ventrolateral tegmentum. Small biocytin crystals (B4261, Sigma, St. Louis, MO) were then placed in the lesioned area until they dissolved completely (about 10 min). The injection site was then washed thoroughly with Ringer’s and the preparations were kept overnight in Ringer’s to allow the tracer to diffuse passively in the axons. The next day, the preparations were fixed by immersion in 4% paraformaldehyde in PBS for 24 h and cut transversally with a cryostat (see above). The next day, the sections were incubated for 1 h with streptavidin conjugated to Alexa Fluor 488 (Invitrogen, S11223) diluted 1:500 in PBS to reveal the presence of biocytin. The slides were then mounted with Vectashield containing DAPI (4′,6-diamidino-2-phenylindole) to label DNA contained in cell nuclei (H-1200, Vector Laboratories).

A series of experiments was designed to identify and localize candidate neurons in the caudal hindbrain that would be involved in the generation of the slow rhythm. The experiments were performed in a whole-brainstem preparation (hindbrain and mesencephalon) isolated *in vitro*, so that both the fast and the slow rhythm generators were included. Axonal tracers were injected in the pTRG and in the lateral tegmentum in the caudal hindbrain. The latter injection was made either on the same or the opposite side to the injection in the pTRG. Candidate neurons of the slow rhythm generator projecting to these target structures were then mapped on the side opposite to the lateral tegmentum injection in the caudal hindbrain.

**FIGURE 1:**
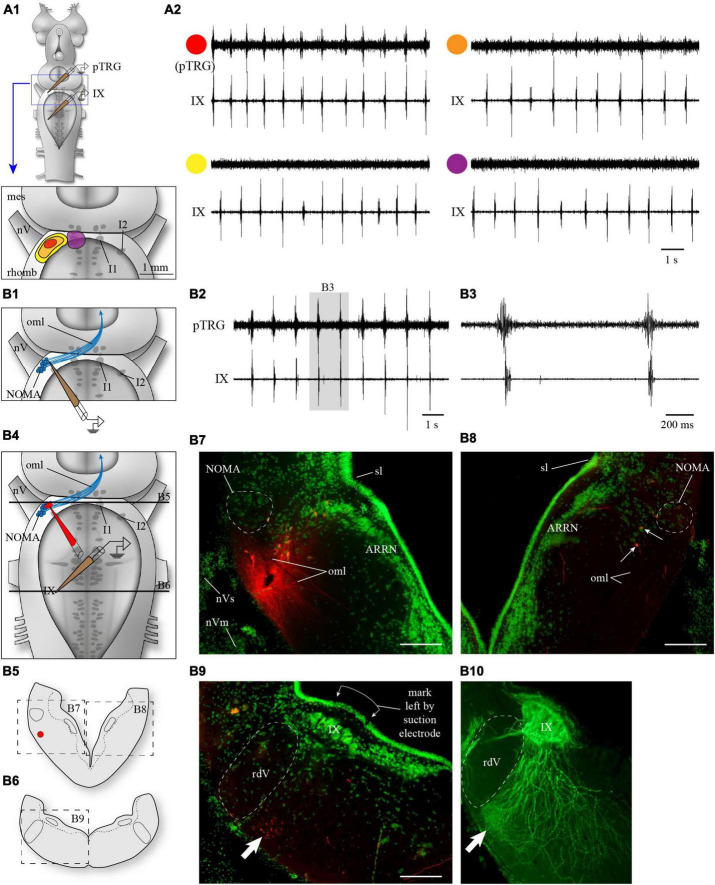
Anatomical localization of the fast rhythm generator, the pTRG, in the rostral hindbrain by electrophysiology and marker injections. **(A1)** The rostral hindbrain was probed from its dorsal aspect with an extracellular electrode to locate the region with the optimal pTRG signal. The search focused on the area around cell I1 (violet in **A1**), which was visualized by cutting open the dorsal midline portion of the isthmus. No areas around cell I1 showed any rhythmical activity (**A2**, trace with violet circle). On the other hand, a clear signal (**A2**, trace with red circle) was easily and reproducibly obtained when the electrode was lowered underneath the alar plate, following a somewhat steep angle through the sulcus limitans aiming at more ventral levels, in the area rostro-lateral to the trigeminal motor nucleus (red spot in **A1**). This optimal area was relatively small, and slightly moving the electrode away from this area in all directions saw the signal decrease (orange in **A1,A2**) or disappear altogether (yellow in **A1,A2**). All recordings shown in panel **(A2)** are from the same animal. **(B1)** Anatomical localization of the area generating optimal pTRG signal in the transverse plane in one example animal. An electrode was lowered only once aiming at the location represented by the red spot in **A1**, at a depth where the pTRG signal was optimal **(B2)**. The signal obtained in this area preceded the respiratory bursts recorded from glossopharyngeal motoneurons **(B3)**. The electrode was removed and quickly replaced with an injection micropipette of the same size filled with Texas Red-dextran amines **(B4)**. The injection micropipette was lowered with the same angle through the visible hole left in the tissue by the recording electrode, until it reached the same depth. A single puff of dextran amines was then delivered to mark the spot. The red labeling left by the dextran amines can be seen **(B7)** on a cross section of the rostro-lateral hindbrain (**B5**, at the level of the top black line in **B4**) near a population of neurons that picked up the dye. Some axonal tracing occurred and neurons on the contralateral side were labeled, in a location corresponding to that of the neurons labeled on the injected side (**B5**, and arrows in **B8**). More caudally, on a cross section at the level of the recording from IX motoneurons (**B6**, at the level of the bottom black line in **B4**), many labeled axons were seen in the lateral tegmentum, most found near the ventral surface (arrow in **B9**), where respiratory motoneurons show a dense arborization of their distal dendrites (arrow in **B10**). The motoneurons in **B10** were labeled after an injection of biocytin (green) in the glossopharyngeal nerve in the periphery (different animal). The green labeling in **B7–B9**, is a fluorescent Nissl staining. ARRN, anterior rhombencephalic reticular nucleus; I1, isthmic cell 1; I2, isthmic cell 2; IX, glossopharyngeal motor nucleus; mes, mesencephalon; NOMA, nucleus octavomotor anterior; nV, trigeminal nerve; nVm, trigeminal motor nerve; nVs, trigeminal sensory nerve; oml, lateral octavo-mesencephalic tract; pTRG, paratrigeminal respiratory group; rdV, descending root of the trigeminal nerve; rhomb, rhombencephalon; sl, sulcus limitans. Scale bars in photomicrographs = 200 μm.

For pTRG tracer injections, the tip of a pulled glass micropipette was used to make a small incision in the rostro-lateral hindbrain around cell I2, in the site where the extracellular signal of the fast rhythm was previously shown to be the strongest [refer to Figure 8 in Martel et al. (2007) and to Figures 1, 2 in Gariépy et al. (2012a)]. Biocytin crystals were placed in the lesioned area until they dissolved completely (about 10 min). For the lateral tegmentum injections, a microsurgical knife (#10318-14, Fine Science Tools, Foster City, CA) was used to make a longitudinal incision in the tegmentum along the medial edge of the respiratory motoneurons of the VII, IX and X motor nuclei. The depth of the incision was about 2/3 of the tegmentum thickness. The goal was to retrogradely label commissural neurons on one side of the brain by cutting (and labeling) their axon in the lateral tegmentum on the opposite side, near the respiratory motoneurons and their extensive ventrolateral dendritic tree (Gariépy et al., 2012a). Crystals of Texas Red-dextran amines (Invitrogen, D3328, 3000 MW) were then placed in the incision until they dissolved completely. In all cases, the pTRG injection was carried out first and special care was taken to make sure that no tracer from the second injection would contaminate the site of the first injection. Each injection site was washed thoroughly with Ringer’s and the preparations were kept overnight in Ringer’s to allow tracer diffusion. The next day, the preparations were fixed by immersion in 4% paraformaldehyde and processed the same way as described above. The biocytin was revealed with streptavidin conjugated to Alexa Fluor 488 and the slides were mounted.

### 2.4 Data acquisition and analyses

#### 2.4.1 Anatomy

All sections were examined using an Eclipse E600 epifluorescence microscope (Nikon Canada, Mississauga, Ontario) and photographed with a DXM1200 digital camera and control software (Nikon). The photomicrographs were adjusted for levels to increase contrast and some of them were superimposed by importing them onto different layers in Photoshop (v.CS5, Adobe) and using the “screen” function to make all layers visible at the same time. The resulting superimposition was then exported as an image.

The detailed location of the candidate neurons for the slow respiratory rhythm generator labeled with the tracers in the caudal hindbrain was examined. Only the side opposite to the lateral tegmentum injection was analyzed because the background fluorescence was too intense on the injected side to identify individual neurons reliably. Using Illustrator software (v.CC, RRID:SCR_010279, Adobe Systems Canada, Ottawa, Ontario), cell bodies were represented by dots positioned directly on imported photograph of the cross sections, and then confirmed back under the microscope, using the DAPI stain to make sure of the presence of a cell nucleus. The structures involved in the analysis included the whole tegmentum plus the descending root of the trigeminal nerve (rdV) and the area close to the sulcus limitans. Numerous neurons were found throughout the octavolateral area, but they were excluded from the analysis because previous studies have shown that they do not play a role in respiratory rhythmogenesis. Indeed, this area contains populations of neurons associated with the vestibular and lateral line systems (Pflieger and Dubuc, 2000, 2004) and chemical manipulation of excitatory or inhibitory transmission in that region does not affect respiratory activity *in vitro* (Cinelli et al., 2016).

The caudal hindbrain was reconstructed in 3D for two animals presenting typical results that were obtained from two different sets of injections (injections on the same side; injections on opposite sides). The contour of one in every three sections of the caudal hindbrain and the location of the labeled neurons were drawn using Illustrator software (Adobe). The drawings were then imported into AutoCAD (v.2014, RRID:SCR_021072, Autodesk Canada, Montréal, Québec) and converted into 3D objects that were later imported back into Illustrator as images for figure assembly and design.

#### 2.4.2 Physiology

Data were acquired using a Digidata 1322A interface using Clampex 9.2 software (pClamp suite, RRID:SCR_011323, Axon Instruments) for computer analysis. The slow rhythm bursts could easily be distinguished from the fast rhythm bursts, because they were approximately 10 times longer (Martel et al., 2007). They were then manually detected using Spike2 version 5.19 (RRID:SCR_000903, Cambridge Electronic Design, Cambridge, UK). The slow rhythm occurred as episodes of single or multiple successive bursts. When the interval between the bursts exceeded 3 s in juvenile lampreys or 10 s in adult lampreys, the following event was considered as a new episode. The same situation occurred when a fast rhythm burst interrupted two slow rhythm bursts. When analyzing effects on cycle durations, a minimum of 10 cycles was always used.

To examine the effects of electrical or chemical (D,L-Glutamate) stimulation, the number of slow rhythm bursts was measured 5 s before and after the start of an electrical stimulation, and 10 s before and after the start of a chemical stimulation. A total of 10 (electrical) and 8 (D,L-glutamate) stimulations were analyzed for the three sites in each animal.

Data in the text and figures are given as means ± standard deviation. Statistical analyses were carried out using SigmaPlot v12.5 (RRID:SCR_003210, Systat Software). A paired *t*-test or a one-way ANOVA for repeated measures was used to test for statistical significance of changes in the measured data. When normality failed, a Wilcoxon signed rank test, or a one-way ANOVA for repeated measures on ranks was used. *Post hoc* pairwise comparisons were performed using the Holm-Sidak or the Student-Newman-Keuls multiple comparisons tests. The differences were considered statistically significant when the *P*-value was equal to or lower than 0.05.

## 3 Results

### 3.1 On the location of the fast respiratory rhythm generator

As mentioned in the introduction section, the fast respiratory rhythm generator in lampreys has traditionally been located at the rostro-lateral pole of the trigeminal motor nucleus near cell I2. More recently, this view was challenged by Cinelli et al. (2013) when they suggested that the fast respiratory rhythm generator was located more rostrally and medially, in an area dorsal to cell I1. Because of these conflicting results, we first re-examined the location of the fast rhythm generator using physiological and anatomical experiments.

In a first set of experiments (*n* = 14), extracellular recordings were performed in the same preparation in the vicinity of cell I1 and the whole area lateral to the trigeminal motor nucleus, especially the area close to cell I2 (Figure 1A1). Recordings at the level of cell I1 revealed no respiratory-related activity (Figures 1A1, A2, violet), whereas recordings closer to the area around cell I2 revealed large respiratory bursts synchronized with respiration recorded from the IX motor nucleus (Figures 1A1, A2, red), as previously shown in Martel et al. (2007, Figure 8). Moving the recording electrode in any direction away from cell I2 caused a clear gradual decrease in the signal amplitude (Figures 1A1, A2, orange) until no signal could be distinguished from the background noise (Figures 1A1, A2, yellow). The absence of activity in the vicinity of cell I1 clearly indicates that generator neurons are not located in that area. On the other hand, the presence of rhythmic activity around cell I2 suggests that rhythmogenic neurons could be located in that area. It should be kept in mind that the presence of rhythmic activity does not necessarily mean that the neurons are rhythmogenic.

In some brainstem preparations, a single penetration of the recording electrode aiming at the pTRG (red spot in Figure 1A1) close to cell I2 immediately provided a clear signal of the fast respiratory rhythm. Five of those preparations were used specifically to locate more precisely the site of such recordings on histological cross-sections. Figures 1B2, B3 show the recordings obtained from one of these animals. The recording electrode was then quickly removed and replaced with an injection electrode filled with a tracer. The tracer was delivered at the spot where the recording was made (Figures 1B4, B5, B7, see Methods section). Some transport of the tracer occurred and retrogradely labeled cells were found (small arrows in Figure 1B8) on the contralateral side in a location corresponding to the site of the ipsilateral injection. In the same animal, fibers that descended in the ventrolateral tegmentum were also labeled. They reached the caudal levels from which the respiratory motoneurons were recorded and were particularly numerous in the most ventrolateral aspect of the tegmentum (Figure 1B6 and arrow in Figure 1B9), an area where a very dense dendritic arborization of the respiratory motoneurons is present (arrow in Figure 1B10).

**FIGURE 2:**
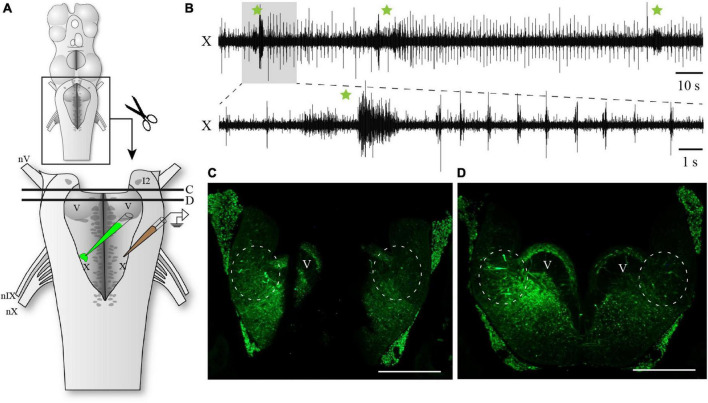
Respiratory activity in the hindbrain after removal of the mesencephalon and the medial portion of the isthmus. **(A)** Schematic drawing illustrating the isolated hindbrain preparation with the removal of the medial isthmic region comprising cell I1 and surrounding structures. The respiratory activity was recorded with an extracellular electrode placed over the X motor nucleus. The illustration also indicates the location where biocytin was injected after the electrophysiological experiment. Black lines labeled C and D correspond to the levels of transverse sections photographed in panels **(C,D)**. **(B)** Neurographic recording of the respiratory rhythm from the vagal motor nucleus showing the persistence of both the fast and slow respiratory rhythms in the absence of the mesencephalon and medial isthmic region. The burst episodes of the slow respiratory rhythm are indicated by green stars. The lower trace corresponds to the shaded area in the upper trace. **(C)** Photomicrograph of a transverse section at the most caudal level of the medial lesion in the rostral hindbrain. Circles indicate the presence of neurons projecting to the vagal motoneurons in the intact lateral areas at this level, close to the sulcus limitans. **(D)** Photomicrograph of a transverse section just caudal to the medial rostral lesion showing the intact hindbrain at mid-levels of the trigeminal motor nucleus. Circles indicate the presence of neurons projecting to the vagal motoneurons in the lateral areas at this level, close to the sulcus limitans. I2, isthmic cell 2; V, trigeminal motor nucleus; X, vagal motor nucleus; nV, trigeminal nerve; nIX, glossopharyngeal nerve; nX, vagal nerve. Scale bars in photomicrographs = 500 μm.

To determine if the region around cell I1 was necessary for the generation of the fast rhythm, we completely removed the mesencephalon and the medial portion of the rostral hindbrain comprising cell I1 (Figure 2A). In these preparations, the fast respiratory rhythm was still present in the respiratory motoneurons (Figure 2B; *n* = 5). The first part of the video recording presented as Supplementary material shows a semi-intact preparation in which most of the spinal cord (caudal complete transection), the mesencephalon and prosencephalon (rostral complete transection) have been removed. In this preparation, the fast and slow respiratory rhythms were both present and similar to what is observed in intact animals. In the second part of the video (recorded from the same preparation), the medial portion of the isthmic region comprising cell I1 has been removed. The fast and slow respiratory rhythms were still present, although the frequency of the fast rhythm was slower. At the end of each of these experiments, the vagal motor nucleus was injected with the axonal tracer, biocytin, and transverse sections of what remained of the rostral hindbrain were examined in search for neurons that projected to the respiratory motoneurons. In each case, it was confirmed that the medial portion of the rostral hindbrain had been completely removed (example in Figure 2C) and that labeled neurons were present in the remaining rostro-lateral rhombencephalon close to cell I2 (circled areas in Figures 2C, D).

**FIGURE 3:**
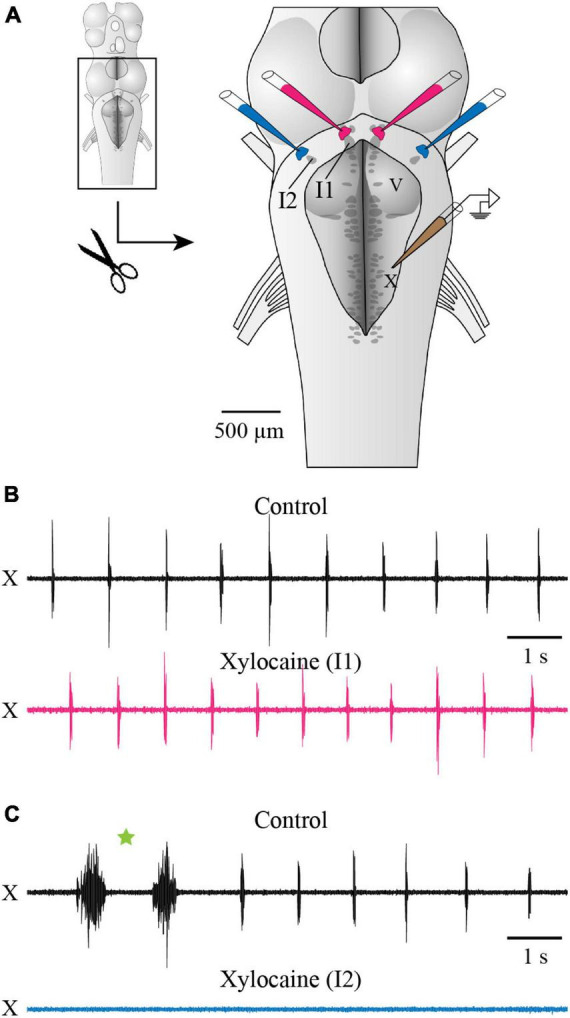
Effects of Xylocaine injections over cells I1 and I2 on respiratory activity recorded from the vagal motor nucleus. **(A)** Schematic drawing of the isolated brainstem *in vitro* preparation showing the localization of the bilateral injections of Xylocaine around cells I1 and I2, as well as the position of the extracellular electrode placed over the X motor nucleus. A dorsal midsagittal section was performed at the level of the isthmus (not illustrated), to make sure cell I1 was completely visible for better pipette positioning. **(B,C)** Neurographic traces illustrating the respiratory activity recorded from the X motor nucleus under control conditions [top traces in panels **(B,C)**] and following the bilateral injection of Xylocaine over cell I1 area [bottom trace in panel **(B)**] and over cell I2 area [bottom trace in panel **(C)**]. One double-burst episode of the slow rhythm is indicated by a green star in panel **(C)**. The pipettes aiming at cell I1 area were positioned on the ependymal surface of the ventricle immediately dorsal to cell I1, while the ones aiming at cell I2 area were lowered through the sulcus limitans underneath the alar plate, as illustrated in Figures 1B1, B4. Note that respiratory activity is abolished when Xylocaine is injected over cell I2 area, but not when injected over cell I1 area. I1, isthmic cell 1; I2, isthmic cell 2; V, trigeminal motor nucleus; X, vagal motor nucleus.

Another series of experiments was designed to compare the effects of inactivating chemically and reversibly the areas around cell I1 and cell I2, in the same preparation (Figure 3A). Bilateral micro-injections of Xylocaine just dorsal to cell I1 did not significantly affect the fast respiratory rhythm frequency (89.17 ± 23.37% of control; paired *t*-test, *P* = 0.084). On the other hand, bilateral micro-injection of Xylocaine in the region of cell I2 completely abolished the fast respiratory rhythm for an average of 4.3 ± 2 min (Figures 3B, C). The fast rhythm was not stopped by injecting unilaterally around cell I2, but it was stopped within a few seconds by an additional injection to the other side. The fast rhythm bursts eventually recovered to values near the control condition (103.8% of control; paired *t*-test, *P* = 0.429, *n* = 8 injections in 5 animals).

**FIGURE 4:**
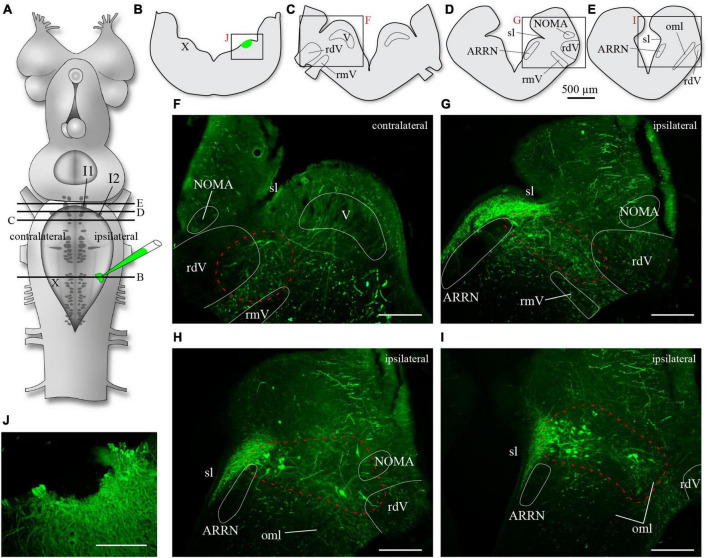
Neurons retrogradely labeled in the rostral hindbrain after a superficial injection of biocytin in the population of motoneuronal cell bodies of the rostral vagal motor nucleus. **(A)** Schematic drawing of the brain of an adult lamprey showing the location of the axonal tracer injection in the vagal motor nucleus. The black lines labeled from B to E refer to the level of sectioning of transverse sections illustrated in B to E. The red letters next to the frames in panels **(B–E)** refer to the photomicrographs in panels **(F–J)**. In all photomicrographs from panels **(F–J)**, the green labeling is biocytin revealed with streptavidin-Alexa Fluor 488. The photomicrograph shown in panel **(H)** is of a transverse section at the level between that of panels **(D,E)**, or in other words, at a level between panels **(G,I)**. Areas including retrogradely labeled neurons are delimited by a red dashed line in panels **(F–I)**. Retrogradely labeled neurons are found both close to cells I2 [panel **(G)** is just rostral to the level of cell I2] and I1 [panel **(I)** is just rostral to the level of cell I1]. ARRN, anterior rhombencephalic reticular nucleus; I1, isthmic cell 1; I2, isthmic cell 2; NOMA, nucleus octavomotor anterior; oml, lateral octavo-mesencephalic tract; rdV, descending root of the trigeminal nerve; rmV, motor root of the trigeminal nerve; sl, sulcus limitans; V, trigeminal motor nucleus; X, vagal motor nucleus. Scale bars in photomicrographs = 200 μm.

We also examined the issue anatomically by injecting a retrograde axonal tracer within the vagal motor nucleus. Such experiments had already been carried out by us in the past (Gariépy et al., 2012a,b) where we had shown that tracer injections in the superficial cell body layer or in the deeper distal dendrites of motoneurons both yielded retrogradely labeled neurons around cells I1 and I2, therefore including parts of the MLR of lampreys. In Cinelli et al. (2013), the injections were located in the superficial motoneuronal cell body layer and they found neurons exclusively around cell I1. To replicate these results, animals were injected in the superficial cell layer of the X motor nucleus (Figure 4, *n* = 3). In 3 other animals, the injections also included the deeper areas of the lateral tegmentum, about halfway between the dorsal and the ventral surfaces. In one other preparation, the injection included even deeper areas, reaching the most distal motoneuronal dendrites in the ventrolateral tegmentum (see white arrows in Figures 1B9, B10). In all animals, neurons were labeled in a location dorsal to cell I1 on both sides (Figures 4H, I), and neurons were also labeled bilaterally in the lateral area along the rostral half of the trigeminal motor nucleus and more rostrally (Figures 4F–I), an area closer to cell I2 that has been associated with the region of the fast rhythm generator (Martel et al., 2007).

In summary, the data in this section confirm that the fast respiratory rhythm is generated by a population of neurons in the area lateral to the trigeminal motor nucleus, with a clear focus point located around cell I2 and areas ventrolateral to it. We also show that the population of neurons located dorsal to cell I1, although projecting to the respiratory motoneurons, are not necessary for the generation of the fast respiratory rhythm in lampreys. These neurons are located in an area previously described as part of the MLR in lampreys (Brocard and Dubuc, 2003; Le Ray et al., 2003; reviewed in Ryczko and Dubuc, 2013).

### 3.2 On the location of the slow respiratory rhythm generator

#### 3.2.1 Anatomical tracing experiments

**FIGURE 5:**
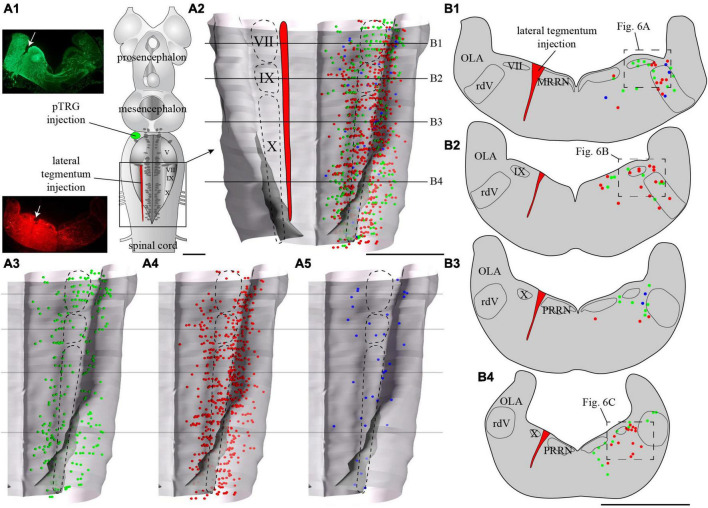
Localization of neurons in the caudal hindbrain (OLA excluded) projecting to the pTRG and the lateral tegmentum on the other side: both injections were made on the same side of the brain. **(A1)** Schematic drawing of a dorsal view of the whole lamprey brain indicating the location of the injection sites. To the left, photomicrographs illustrating the injection sites on transverse sections for the animal represented in the figure. **(A2)** 3D rendering of the caudal hindbrain with neurons projecting exclusively to the pTRG in green, exclusively to the lateral tegmentum on the other side in red, and projecting to both in blue. The location of the respiratory VII, IX, and X motor nuclei is indicated by dashed lines. **(A3)** Representation of the neurons that project exclusively to the pTRG as they appear in panel **(A2)**. **(A4)** Representation of the neurons that project exclusively to the lateral tegmentum on the other side as they appear in panel **(A2)**. **(A5)** Representation of the neurons that project to both the contralateral pTRG and contralateral tegmentum as they appear in panel **(A2)**. **(B1–B4)** Drawings of transverse sections of the caudal hindbrain at the levels indicated on the 3D rendering. The color code is the same as in **A**. IX, glossopharyngeal motor nucleus; MRRN, middle rhombencephalic reticular nucleus; OLA, octavolateral area; PRRN, posterior rhombencephalic reticular nucleus; pTRG, paratrigeminal respiratory group; rdV, descending root of the trigeminal nerve; VII, facial motor nucleus; X, vagal motor nucleus. Scale bars = 1 mm.

To characterize the distribution of the candidate neurons generating the slow respiratory rhythm, dextran amines conjugated to Texas Red were injected in the lateral tegmentum along the respiratory motoneuron pools (VII, IX, X) in the caudal hindbrain, whereas biocytin was injected in the contralateral (*n* = 3) or ipsilateral (*n* = 3) pTRG. The lateral tegmentum injections labeled neurons on the opposite side along the rostro-caudal extent of the caudal hindbrain, in the medial and lateral tegmentum near the VII, IX, and X respiratory motor nuclei. Most of these neurons were exclusively labeled by the lateral tegmentum injection and are illustrated as red dots in Figures 5, 7. Some neurons from this population were also found in the rdV as well as in an area close to the sulcus limitans. The lateral tegmentum injections were made deep in the tissue to ensure the labeling of the largest amount of crossing axons (Figures 5A1, B, 7A1, B). As indicated in the Methods section, the numerous labeled neurons in the octavolateral area were not included in the analysis.

**FIGURE 6:**
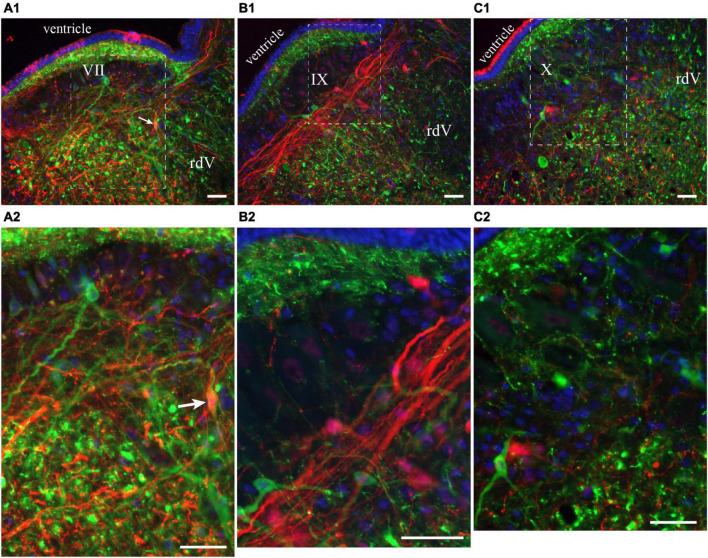
Neurons in the lateral tegmentum of the caudal hindbrain were retrogradely labeled after tracer injections in the contralateral pTRG and the lateral tegmentum on the other side (see Figure 5A1). **(A1,B1,C1)** Photomicrographs of neurons projecting to the contralateral pTRG (green), to the contralateral lateral tegmentum (red) and to both [white arrows in panels **(A1,A2)**]. Refer to Figures 5B1, B2, B4 for the approximate location of the photographic frames. Panels **(A2,B2,C2)** are enlargements of the areas delineated by a white dashed line in panels **(A1,B1,C1)**, respectively. The blue labeling was obtained with DAPI, which stains DNA in cell nuclei. rdV, descending root of the trigeminal nerve; VII, facial motor nucleus; IX, glossopharyngeal motor nucleus; X, vagal motor nucleus. Scale bars = 50 μm.

**FIGURE 7:**
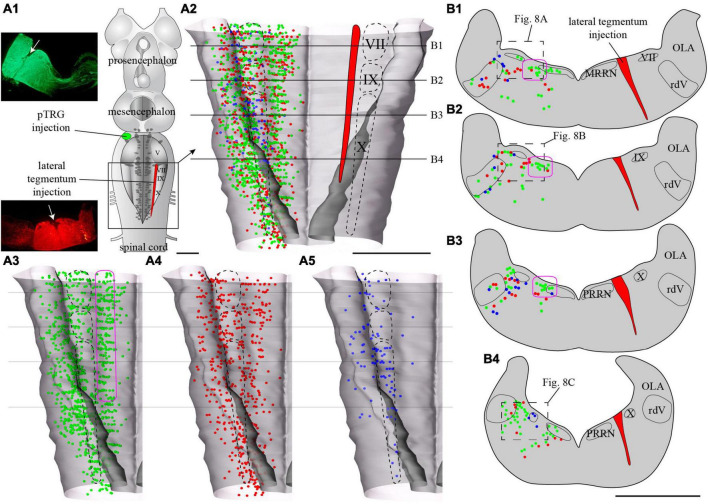
Localization of neurons in the caudal hindbrain (OLA excluded) projecting to the ipsilateral pTRG and the lateral tegmentum on the other side: the two injections were made on opposite sides of the brain. **(A1)** Schematic drawing of a dorsal view of the whole lamprey brain indicating the location of the injection sites. To the left, photomicrographs of the injection sites on transverse sections from the animal represented in the figure. **(A2)** 3D rendering of the caudal hindbrain with neurons projecting exclusively to the pTRG in green, exclusively to the lateral tegmentum on the other side in red, and projecting to both in blue. The location of the respiratory VII, IX, and X motor nuclei is indicated by dashed lines. **(A3)** Representation of the neurons that project exclusively to the pTRG as they appear in panel **(A2)**. **(A4)** Representation of the neurons that project exclusively to the lateral tegmentum on the other side as they appear in panel **(A2)**. **(A5)** Representation of the neurons that project to both the ipsilateral pTRG and contralateral tegmentum as they appear in panel **(A2)**. The purple shape in panels **(A3,B1–B3)**, delimits a conspicuous population of neurons with larger cell bodies and ipsilateral projections to the pTRG region. Examples can be seen in Figure 8B1. More details are available in the text. **(B1–B4)** Drawings of transverse sections of the caudal hindbrain at the levels indicated on the 3D rendering in panels **(A2–A5)**. The color code is the same as in panel **(A)**. IX, glossopharyngeal motor nucleus; MRRN, middle rhombencephalic reticular nucleus; OLA, octavolateral area; PRRN, posterior rhombencephalic reticular nucleus; pTRG, paratrigeminal respiratory group; rdV, descending root of the trigeminal nerve; VII, facial motor nucleus; X, vagal motor nucleus. Scale bars = 1 mm.

**FIGURE 8:**
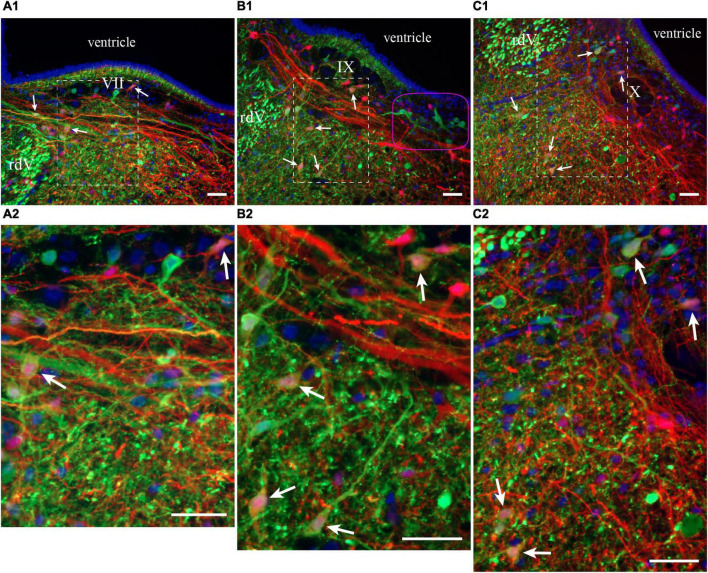
Neurons in the lateral tegmentum of the caudal hindbrain were retrogradely labeled after tracer injections in the ipsilateral pTRG and the lateral tegmentum on the other side (see Figure 7A1). **(A1,B1,C1)** Photomicrographs of neurons projecting to the pTRG (green), to the lateral tegmentum on the other side (red), and to both (white arrows). Refer to Figures 7B1, B2, B4 for the approximate location of the photographic frames. The purple shape in B1 refers to the corresponding area in Figures 7A3, B1–B3. Panels **(A2,B2,C2)** are enlargements of the areas delineated by a white dashed line in panels **(A1,B1,C1)**, respectively. The blue labeling was obtained with DAPI, which stains DNA in cell nuclei. rdV, descending root of the trigeminal nerve; VII, facial motor nucleus; IX, glossopharyngeal motor nucleus; X, vagal motor nucleus. Scale bars = 50 μm.

Tracer injections in the pTRG also retrogradely labeled neurons in the caudal hindbrain ipsi- and contralateral to the injection. As was the case for the lateral tegmentum injections, labeled neurons projecting to the pTRG were found in proximity to the respiratory motor nuclei along the rostro-caudal extent of the lateral tegmentum. Most of these neurons were exclusively labeled by the pTRG injection and are illustrated as green dots in Figures 6, 8. Some neurons from this population were also observed in the rdV and close to the sulcus limitans. In addition, a population of larger neurons was found ipsilateral to the pTRG injection, between the respiratory motoneurons and the more medial reticulospinal cells, at the levels of the posterior rhombencephalic reticular nucleus and the caudal part of the middle rhombencephalic reticular nucleus (purple shapes in Figures 7A3, B1–B3, 8B1). These larger neurons only projected to the ipsilateral pTRG. In general, the distribution of the labeled neurons in the caudal hindbrain was very similar on both sides, but a larger number of neurons was observed on the side of the pTRG injection (compare green dots in Figures 5A3, 7A3). Control experiments were carried out in which the animals received a single tracer injection in the pTRG. A larger number of neurons were also labeled on the side ipsilateral to the injection (*n* = 3) in these control experiments.

Of particular interest in the context of the present study, many neurons were labeled by both injections, whether these injections were ipsilateral (*n* = 3) or contralateral (*n* = 3) to each other. The double-labeled neurons were illustrated in Figures 5, 7 as blue dots, but were not illustrated as red or green dots because we voluntarily wanted to make this population of neurons stand out. They were observed across the rostro-caudal extent of the lateral tegmentum in the caudal hindbrain, with more of them on the side of the pTRG injection (compare blue dots in Figures 5A5, 7A5).

Overall, these anatomical results revealed that the lateral tegmentum of the caudal hindbrain contains numerous candidate neurons that could be involved in the generation of the slow rhythm and modulation of the fast rhythm. The neurons were distributed longitudinally, and many were in proximity with the respiratory motoneurons. Some projected to the fast rhythm generator area (pTRG), others projected to the lateral tegmentum on the opposite side, and others projected to both.

#### 3.2.2 Effects of electrical and chemical stimulation on the slow rhythm generator

**FIGURE 9:**
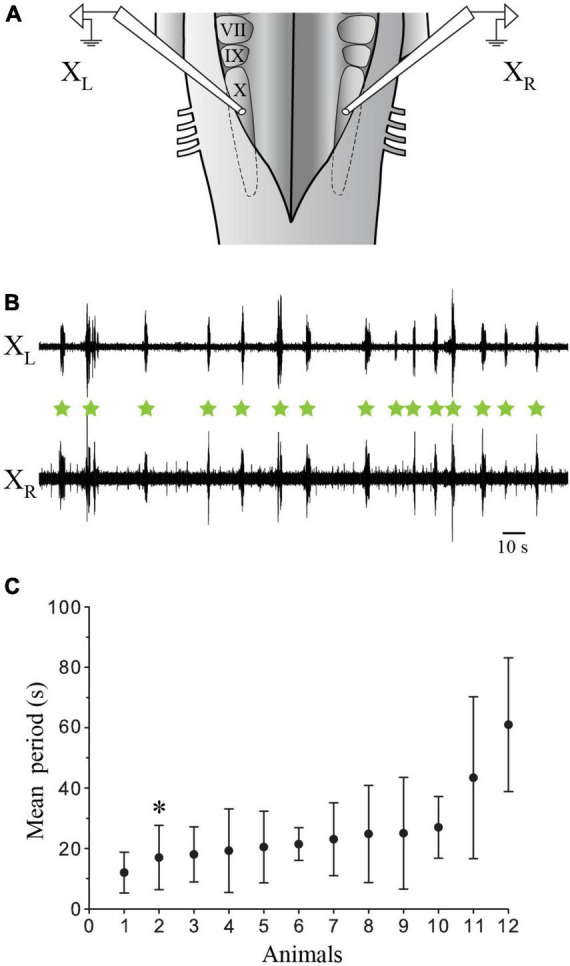
Extracellular recordings of the slow rhythm in the isolated caudal hindbrain preparation. **(A)** Illustration of the *in vitro* isolated caudal hindbrain showing the localization of the extracellular electrodes over the respiratory motoneurons on both sides. **(B)** Neurographic traces illustrating many burst episodes (green stars) of the slow rhythm recorded simultaneously from the left (L) and right (R) X motor nuclei. Note that the fast rhythm is absent. **(C)** Graph illustrating the mean period (s) of the slow rhythm in 12 animals. The neurographic traces in B are from animal 2 (*). VII, facial motor nucleus; IX, glossopharyngeal motor nucleus; X, vagal motor nucleus.

Physiological experiments were carried out in the *in vitro* isolated caudal hindbrain preparation to characterize the putative role of the labeled neurons (Figures 5–8) in generating the slow respiratory rhythm. In this preparation, only the slow rhythm is still present and can be recorded from the respiratory motoneurons (Figure 9). The raw traces in Figure 9B illustrate the bilaterally synchronized slow rhythm recorded extracellularly on the surface of the X motor nucleus on both sides (mean period 26.04 s ± 6.31 s, ranging from 3.36 s to 96.77 s; *n* = 12; Figure 9C). The period tended to display large variability between animals and life stages (see last paragraph of the Results section).

**FIGURE 10:**
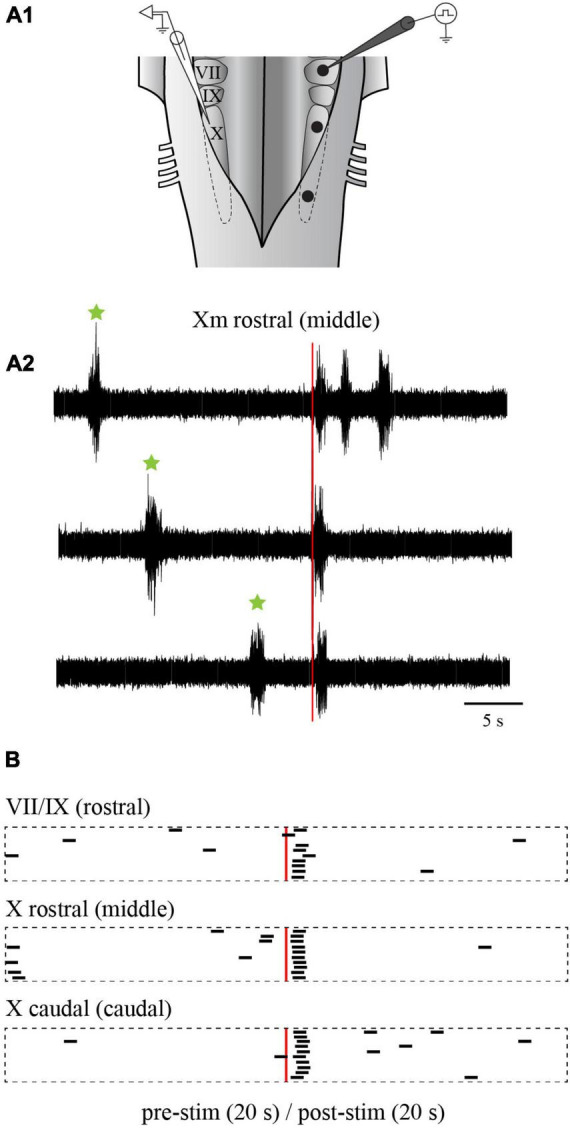
Effects of electrical stimulation of the lateral tegmentum in the isolated caudal hindbrain preparation. **(A1)** Illustration of the caudal hindbrain preparation showing the localization of the extracellular recordings and the three stimulation sites. **(A2)** Neurographic traces showing three examples of slow rhythm bursts triggered by the stimulation (red vertical line) of the middle site. Spontaneous slow rhythm burst episodes preceding the stimulation are indicated by green stars. **(B)** Peristimulus histograms (20 s before and after the stimulation) illustrating the occurrence of extracellular bursts typical of the slow rhythm recorded over the X motor nucleus on one side, following electrical stimulations (3 pulses of 9 μA, 2 ms at a frequency of 30 Hz every 50 s) in the VII and IX motor nuclei (rostral site), the rostral X motor nucleus (middle site) and the caudal X motor nucleus (caudal site) on the opposite side. The histograms are aligned on the stimulation illustrated by the red vertical line. The black bars each represent one slow rhythm burst, with their left end aligned with the onset of the burst. The length of each bar is equal to the mean duration of a triggered burst from the middle site. VII, facial motor nucleus; IX, glossopharyngeal motor nucleus; X, vagal motor nucleus.

We first examined the effects of electrical stimulation of the lateral tegmentum in the caudal hindbrain on the slow rhythm. As indicated in the Methods section, three equidistant stimulation sites (rostral, middle, and caudal) were arbitrarily chosen along the longitudinal axis. Trains of stimulation were delivered in each site in the same animal, while contralateral X motoneuron activity was recorded from the ventricular surface (Figure 10A1). Bursts of discharge were triggered by stimulating any of the three sites (Figure 10A2). The bursts occurred at a similar latency for the rostral (1.09 ± 0.73 s), middle (0.98 ± 0.74 s), and caudal (0.98 ± 0.92 s) sites (one-way ANOVA for repeated measures, *P* = 0.694; Figures 10A2, B). A comparison of spontaneous vs. triggered bursts duration indicated that the triggered bursts had similar characteristics as the spontaneous slow rhythm bursts (0.96 ± 0.53 s vs. 1.02 ± 0.44 s, paired *t*-test, *P* = 0.514). The number of bursts was compared 5 s before and after the stimulation. For the three stimulated sites, the number of bursts was significantly larger following the stimulation (Table 1; *n* = 7), but the difference between the number of triggered bursts from the three sites was not significantly different (one-way ANOVA for repeated measures, *P* = 0.064). The threshold intensity needed to trigger a burst following the stimulation was analyzed for the three sites. It was defined as the intensity needed to generate a burst 50% of the time. The threshold was higher for the rostral site compared to the two other sites (rostral: 5.6 ± 0.82 μA; middle: 3.5 ± 0.87 μA; caudal 3.6 ± 0.55 μA; one-way ANOVA for repeated measures, *P* = 0.004; Holm-Sidak method multiple comparisons test, *P* < 0.05; *n* = 5).

**TABLE 1:** Number of bursts before and after electrical stimulation in the lateral tegmentum.

	5 s pre-stim (total bursts for 70 trials)	5 s post-stim (total bursts for 70 trials)	*p*-value
Rostral	14	59	<0.001
Middle	14	64	<0.001
Caudal	4	64	<0.001

Total number of bursts before and after 70 stimulations per site (*n* = 7). Each site pre- and post-conditions were compared using a Wilcoxon signed rank test.

**FIGURE 11:**
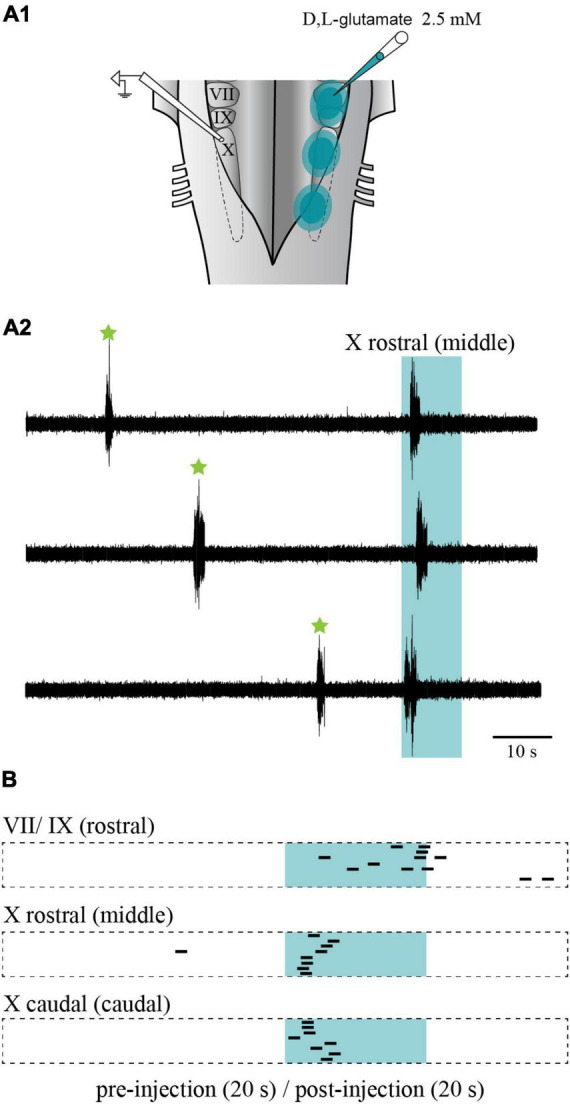
Effects of chemical stimulation of the lateral tegmentum in the isolated caudal hindbrain preparation. **(A1)** Illustration of the caudal hindbrain preparation, showing the localization of the extracellular recordings and the three sites in which D,L-glutamate (2.5 mM) was injected. **(A2)** Neurographic traces showing three examples of slow rhythm bursts during the injection (aqua area) in the middle site. Spontaneous slow rhythm burst episodes before the stimulation are indicated by green stars. The traces are aligned with the beginning of the injection. **(B)** Peristimulus histogram (20 s before and after the stimulation) illustrating the extracellular bursts typical of the slow rhythm recorded over the X motor nucleus, following a series of eight D,L-glutamate injections (5–10 ms pulses at 5 Hz for 10 s every 80 s; 1–3 psi) in the VII and IX motor nuclei (rostral site), the rostral X motor nucleus (middle site) and the caudal X motor nucleus (caudal site) on the opposite side. The histograms are aligned with the injection onset illustrated by the aqua area. The black bars illustrate the onset of the slow rhythm bursts. The length of each bar is equal to the mean duration of a triggered burst from the middle site. VII, facial motor nucleus; IX, glossopharyngeal motor nucleus; X, vagal motor nucleus.

Electrical stimulation was then replaced by chemical stimulation to selectively activate cell bodies in the stimulated area. D,L-glutamate (2.5 mM) was locally injected in the three sites (rostral, middle, and caudal). The injections did not overlap with one another along the rostro-caudal axis, nor did they spread on the contralateral side. Medio-laterally, the injections were contained between the reticulospinal cells and the sulcus limitans (Figure 11A1). Like when electrical stimulation was used, the injection of D,L-glutamate in the three sites triggered bursts that were characteristic of the slow rhythm recorded on the contralateral side (Figures 11A2, B; *n* = 7 sets of injections in 5 animals). The stimulation systematically induced a slow rhythm-like burst. The number of bursts was compared for the 10 s before and after the onset of the injection. As for the electrical stimulation, the number of bursts was significantly larger following the chemical stimulation for every site (Table 2). Stimulation of the middle and the caudal sites generated a larger number of bursts compared to the rostral one (one-way ANOVA for repeated measures, *P* = 0.001).

**TABLE 2:** Number of bursts before and after D,L-glutamate injection in the lateral tegmentum.

	10 s pre-injection (total bursts for 56 trials)	10 s post-injection (total bursts for 56 trials)	*p*-value
Rostral	7	25	<0.001
Middle	9	47	<0.001
Caudal	3	50	<0.001

Total number of bursts before and after 56 stimulations per site (*n* = 7 sets of injections in 5 animals). Each site pre- and post-conditions were compared using a Wilcoxon signed rank test.

Altogether, results from the electrical and chemical stimulation experiments show that the activation of the rostral, middle, and caudal part of the lateral tegmentum in the caudal hindbrain can successfully trigger bursts of discharge that are typical of the slow rhythm, suggesting that the rhythm generating area is distributed rostro-caudally.

#### 3.2.3 Effects of Xylocaine on the slow rhythm generator

To further evaluate the possible contribution of the rostral, middle, and caudal parts of the lateral tegmentum to the generation of the slow respiratory rhythm, we used the isolated caudal hindbrain preparation, this time to inject Xylocaine bilaterally in either of the three sites. Injections of Xylocaine in the rostral (*n* = 6 sets of injection in 4 animals), the middle (*n* = 12 sets of injection in 6 animals), or the caudal (*n* = 5 sets of injection in 5 animals) site abolished the spontaneous slow rhythm for several minutes in all tested animals (Table 3). The amount of time during which the rhythm was abolished (pause duration) was compared for the three stimulation sites. The only significant difference was when comparing the caudal to the rostral site (one-way ANOVA for repeated measures, *P* = 0.03; Holm-Sidak multiple comparison test, *P* < 0.05). In all cases, the slow rhythm recovered after 5–91 min of washout.

**TABLE 3:** Effects of bilateral injection of Xylocaine on the slow rhythm.

	Pause duration (min)	Control period (s)	Washout period (s)	*p*-value
Rostral	27.0 ± 9.8	46.6 ± 39.2	63.5 ± 53.9	=0.004
Middle	18.6 ± 8.4	52.9 ± 43.8	68.9 ± 66.8	= 0.077
Caudal	11.1 ± 9.7	47.6 ± 30.8	74.6 ± 35.4	<0.001

Mean ± SD represent the pause duration (min) and the slow rhythm period (s). For the rostral and middle site, the control and the washout conditions were compared using a Wilcoxon signed rank test. A paired *t*-test was used for the caudal site.

**FIGURE 12:**
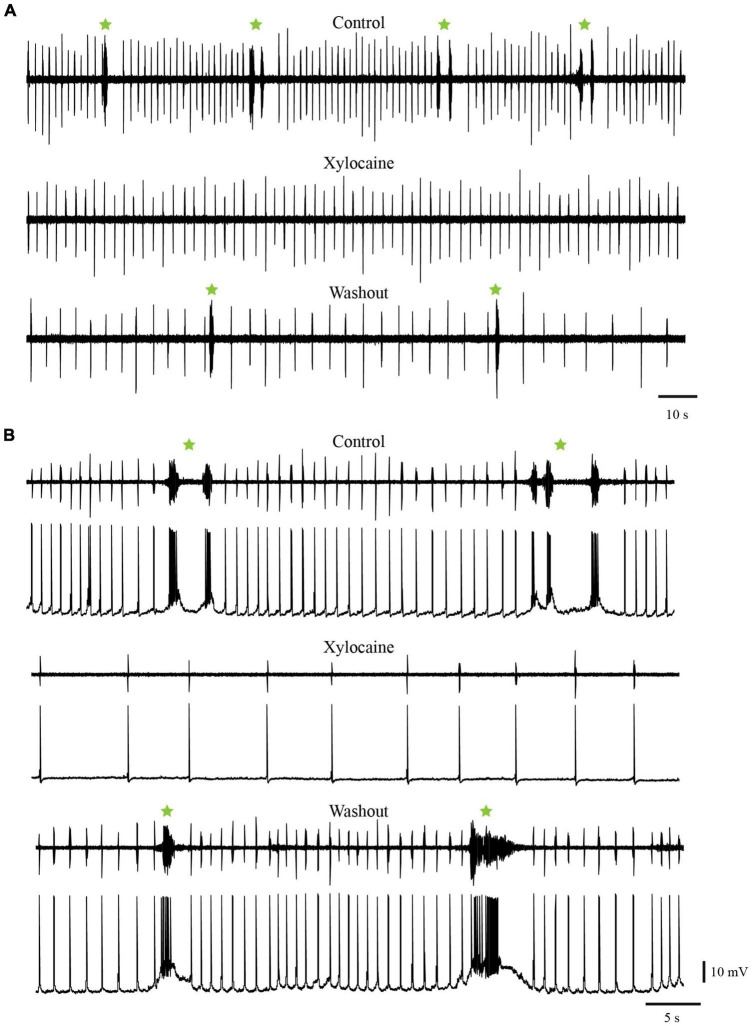
Injections of Xylocaine in the whole-brainstem preparation. The injections were applied bilaterally to the caudal **(A)** or rostral **(B)** tegmental sites and abolished the slow rhythm but not the fast. **(A)** Neurographic traces illustrating respiratory motoneuron activity recorded with an extracellular electrode placed directly over the respiratory motoneurons of the caudal VII/rostral IX motor nuclei. Top: control condition. Middle: after bilateral Xylocaine injection in the caudal site. Bottom: after washout. **(B)** Neurographic traces illustrating respiratory motoneuron activity under control condition (Top), after a bilateral Xylocaine injection in the rostral site (Middle), and after washout (Bottom). Respiration here was recorded with an extracellular electrode placed directly over the respiratory motoneurons in the caudal part of the X motor nucleus (top trace in each condition) and with an intracellular electrode in a respiratory motoneuron located slightly more rostral than the extracellular recording (bottom trace in each condition). The green stars indicate slow rhythm burst episodes, some comprising multiple bursts.

To test if the temporary cessation of the slow rhythm resulted from a decreased excitability of the respiratory motoneurons rather than an effect on generator neurons, the same experiments were repeated in the whole-brainstem preparation, which includes both the fast and slow rhythm generators (Figures 12A, B, control trace). In these experiments, only the rostral and caudal sites were targeted. In the same way as in the experiments performed in the isolated caudal hindbrain preparation, the bilateral injections of Xylocaine in the rostral (*n* = 4 sets of injection across 4 animals) and the caudal (*n* = 6 sets of injection across 5 animals) sites abolished the spontaneous slow rhythm for several minutes in all tested animals (Figures 12A, B Xylocaine trace). There was no significant difference in the pause duration of the slow rhythm between the rostral and the caudal sites (Table 4, *t*-test, *P* = 0.580). The slow rhythm recovered after 13–78 min of washout (Figures 12A, B, Washout trace). Moreover, intracellular recordings of respiratory motoneurons (Figure 12B, bottom traces for all conditions) were carried out in two animals to examine whether there could have been subthreshold slow rhythm membrane potential oscillations still present following a bilateral Xylocaine injection in the rostral site, and it was not the case (Figure 12B, Xylocaine trace).

**TABLE 4:** Effects of bilateral injection of Xylocaine on the slow rhythm in the whole-brainstem preparation.

	Pause duration (min)	Control period (s)	Washout period (s)	*p*-value
Rostral	17.3 ± 11.1	25.4 ± 20.9	47.1 ± 101.5	= 0.024
Caudal	13.7 ± 8.5	47.7 ± 43.1	54.5 ± 49.6	= 0.443

Mean ± SD represent the pause duration (min) and the slow rhythm period (s). For each site the control and the washout conditions were compared using a Wilcoxon signed rank test.

When Xylocaine was injected in the rostral site there was a large reduction in the frequency of the fast respiratory rhythm that could be attributed to the spreading of the drug to the most caudal portion of the fast respiratory rhythm generating region, although this seems unlikely. Since many neurons are projecting from the caudal hindbrain to the fast respiratory rhythm generating region, it is more likely that the injections of Xylocaine in the rostral site could have removed some excitatory ascending inputs (Martel et al., 2007; Cinelli et al., 2016; and present results).

#### 3.2.4 The effects of glutamate antagonists, substance P, and DAMGO on the slow rhythm

**FIGURE 13:**
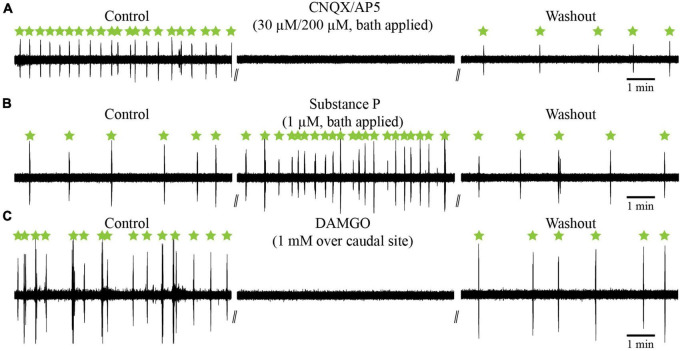
Effects of glutamate antagonists (CNQX/AP5) and neuropeptides (substance P; DAMGO) on the slow respiratory rhythm in the isolated caudal hindbrain preparation. **(A)** Neurographic traces illustrating the slow respiratory rhythm under control, bath-application of glutamatergic antagonists, and washout conditions. **(B)** Neurographic traces illustrating the slow respiratory rhythm under control, bath-application of substance P, and washout conditions. **(C)** Neurographic traces illustrating the slow respiratory rhythm under control, bilateral micro-injections of DAMGO over the caudal site, and washout conditions. All slow rhythm burst episodes are indicated by green stars.

It was shown that the synaptic responses elicited in respiratory motoneurons by stimulation of the fast rhythm generator were blocked by a cocktail of glutamate antagonists, CNQX and AP5, injected over the recorded cell (Gariépy et al., 2012a). To determine whether glutamate transmission is essential for slow rhythm generation, glutamate antagonists (CNQX, 30 μM; AP5, 200 μM) were bath-applied. The drug cocktail abolished the slow rhythm for 29.4 ± 16.9 min on average (Figure 13A; *n* = 2). After washout, the slow rhythm recovered, but with a longer period (78.5 ± 46.0 s vs. 41.5 ± 24.1 s, paired *t*-test; *P* = 0.014).

Vertebrate respiratory generators are sensitive to substance P and to the μ-opioid receptor agonist DAMGO (Bianchi et al., 1995; Gray et al., 1999; Vasilakos et al., 2005; Mutolo et al., 2007, 2010; Chen and Hedrick, 2008; Onimaru et al., 2012). We tested whether the slow rhythm was also sensitive to substance P and DAMGO. Experiments were carried out in the isolated caudal hindbrain preparation. Bath application of substance P significantly reduced the period (19.4 ± 16.3 s) of the slow rhythm compared to the control condition (63.1 ± 64.0 s), with a maximal effect reached 6–24 min after the application of the drug (one-way ANOVA for repeated measures on ranks, *P* < 0.001; Student-Newman-Keuls multiple comparison test, *P* < 0.05; Figure 13B, *n* = 7). After washout, the slow rhythm recovered with a period nearing that of the control condition (78.9 ± 87.7 s vs. 63.1 ± 64.0 s, *P* > 0.05).

The μ-opioid receptor agonist, DAMGO, was then tested. It was injected bilaterally in the rostral (*n* = 5 sets of injection across 5 animals), middle (*n* = 7 sets of injection across 5 animals), and caudal (*n* = 5 sets of injection across 5 animals, Figure 13C) sites. It temporarily abolished the slow rhythm in all tested animals. The rhythm stopped for 27–47 min (see Table 5) and there were no significant differences between the three sites (one-way ANOVA for repeated measures, *P* = 0.487). The slow rhythm recovered after a 47–116 min washout. These experiments suggest that μ-opioid receptors modulate the slow rhythm in the lateral tegmentum of the caudal hindbrain.

**TABLE 5:** Effects of bilateral injections of DAMGO on the slow rhythm.

	Pause duration (min)	Control period (s)	Washout period (s)	*p*-value
Rostral	27.0 ± 15. 6	34.4 ± 25.0	41.1 ± 24.4	= 0.025
Middle	47.2 ± 12.4	43.3 ± 31.3	73.2 ± 57.6	<0.001
Caudal	29.7 ± 9.7	23.1 ± 17.8	32.7 ± 28.3	<0.001

Mean ± SD represent the pause duration (min) and the slow rhythm period (s). For each site the control and the washout conditions were compared. A paired *t*-test was used for the rostral site and a Wilcoxon signed rank test for the others.

**FIGURE 14:**
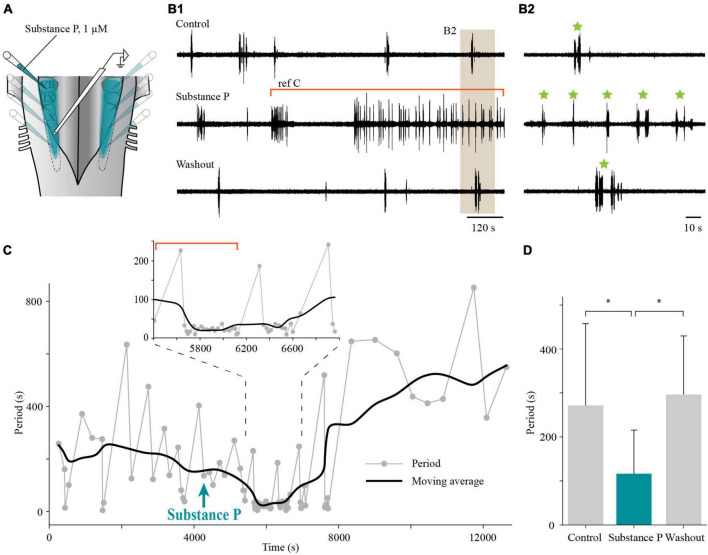
Effects of local application of substance P on the slow respiratory rhythm in the isolated caudal hindbrain preparation of adult lampreys. **(A)** Illustration of the caudal hindbrain preparation showing the localization of the extracellular recording and the area covered by multiple micro-injections of substance P in the lateral tegmentum. **(B1)** Neurographic traces illustrating the slow respiratory rhythm recorded over the X motor nucleus under control, bilateral micro-injections of substance P in the lateral tegmentum, and washout conditions. The segment of the trace representing the substance P condition was chosen to illustrate the gradual effect of the drug. The portion of the trace under the red bracket corresponds to the region of the graph under the red bracket in panel **(C)**. **(B2)** The area shaded in tan in panel **(B1)** was enlarged to reveal episodes (green stars) of single and multiple bursts. **(C)** Graph showing the period of the slow rhythm bursting episodes (gray dots) before and after local, bilateral application of substance P (aqua arrow) in the lateral tegmentum. The black line represents the moving average of the period (average of 10 episodes of bursts). Inset: Enlarged portion of the graph to highlight the higher frequency of burst episodes following local substance P micro-injection. **(D)** Histogram illustrating the mean period during 15 min of control (271.4 ± 186.3 s), local, bilateral injection of substance P in the lateral tegmentum (116.4 ± 98.9 s), and washout (296.1 ± 133.6 s) conditions in six adult lampreys. *P < 0.05.

We then showed that the effects of bath application of substance P described above can be reproduced when locally applied to the lateral tegmentum (Figure 14). In adult lampreys, the period of the slow rhythm was calculated over 15 min during control, substance P, and washout conditions. The period of the slow rhythm was reduced by unilateral or bilateral micro-injections of substance P (116.4 ± 98.9 s; maximal effect reached 16–23 min after application) in comparison to the control condition (271.4 ± 186.3 s; one-way ANOVA for repeated measures, *P* = 0.008; Holm-Sidak multiple comparison test, *P* < 0.05; Figure 14D, *n* = 6) and recovered after washout (296.1 ± 133.6 s; *P* < 0.05; 43–67 min after substance P application). These results suggest that substance P has a general excitatory effect on the slow rhythm generator.

## 4 Discussion

Respiration in lampreys is characterized by a fast and a slow rhythm (Rovainen, 1985; Thompson, 1985; Russell, 1986; Martel et al., 2007; Mutolo et al., 2007, 2010; Gariépy et al., 2012a,b). As indicated previously, the presence of two respiratory rhythms is not a unique feature of basal vertebrates. In most mammals including humans, spontaneous breathing is periodically interrupted by sighs that constitute a slow rhythm (Cherniack et al., 1981). Interestingly in lampreys, the slow rhythm is about 20 to 30 times slower than the fast rhythm and a similar ratio is seen in brainstem slice preparations in mammals. At the cellular level, mammalian respiratory network neurons in the pre-Bötzinger complex generate both the low-frequency, large-amplitude sigh rhythm and the faster, smaller-amplitude eupneic rhythm (Tryba et al., 2008). This indicates that episodes of sighs in mammals do not require sensory inputs to be triggered. Sighing is believed to maintain effective gas exchange by preventing the gradual collapse of alveoli in the lungs. Many studies are presently conducted to define the mechanisms underlying the two respiratory rhythms. Sighs observed in mammals could be homologous to slow rhythm bursts seen in lampreys. One clear distinction, however, is the presence of two distinct sites for the fast and slow respiratory rhythms in lampreys whereas the two rhythms originate in the same site in mammals, in the pre-Bötzinger complex. Further studies are needed to characterize the homology between respiration in mammals and in lampreys.

As mentioned in the introduction, the fast respiratory rhythm in lampreys was shown by a number of independent investigators dating back to the 1980s to originate in the rostral hindbrain, with strong evidence for the implication of a small region lateral to the rostral pole of the trigeminal motor nucleus, near cell I2 (Rovainen, 1985; Thompson, 1985; Russell, 1986; Martel et al., 2007; Gariépy et al., 2012a,b). In contrast, Cinelli et al. (2013) suggested that the fast respiratory rhythm was rather generated in a region identified by us and others as the mesencephalic locomotor region, dorsal to cell I1. Through lesion, physiological, and anatomical experiments, we now show that the region near cell I1 is not necessary for respiratory rhythmogenesis and that the region closer to cell I2 is.

The slow rhythm generator has received much less attention in lampreys (see Missaghi et al., 2016 and Milsom, 2018 for review), although some studies localized it in the caudal hindbrain (Rovainen, 1985; Thompson, 1985; Martel et al., 2007). We now provide further details on the location and the organization of the slow rhythm generator in lampreys. We found that candidate neurons responsible for generating the slow rhythm were distributed longitudinally along the rostro-caudal extent of the caudal hindbrain lateral tegmentum with no obvious clustering. Our physiological experiments show that stimulation along the lateral tegmentum elicits bursts of discharge that are characteristic of the slow rhythm. Conversely, inactivating a part of the longitudinally distributed population of neurons abolishes the slow rhythm altogether. We also show that manipulating glutamate and neuropeptide neurotransmission has powerful effects on the slow respiratory rhythm as described for the respiratory generators in other vertebrate species.

### 4.1 Location of the fast respiratory rhythm generator in lampreys

The fast respiratory rhythm generator, initially known as the rostro-lateral site (RLS; Martel et al., 2007) and later renamed the paratrigeminal respiratory group (pTRG, Mutolo et al., 2007), was physiologically localized many years ago as the region in the rostro-lateral hindbrain where the recorded respiratory signal was the strongest. On transverse sections, it corresponded to an area delimited by cell I2, the anterior octavomotor nucleus (NOMA), and the motor and descending roots of the trigeminal nerve (Russell, 1986; Martel et al., 2007; Mutolo et al., 2007). When looking at a dorsal view of the brainstem, the pTRG is located at the rostro-lateral tip of the trigeminal motor nucleus. As we will see, this location of the pTRG is supported by convincing evidence and the results obtained in the present study add even more strength to it, while demonstrating that the proposed alternative localization of the fast rhythm generator around cell I1 is not compatible with new evidence.

First, the results here confirm that the rostro-lateral hindbrain close to cell I2 shows neuronal activity synchronized with the activity of respiratory motoneurons preceding the latter by tens of milliseconds, which should be expected if these generator neurons are to drive motoneurons. Extracellular recordings may not provide enough resolution to properly assess the timing of the different respiratory events, but previous authors who have recorded from individual local neurons in the pTRG area near cell I2 have also shown a rhythmic activity synchronized with, or preceding, the vagal motoneuron respiratory activity (Russell, 1986; Gariépy et al., 2012a,b). Stimulation of the pTRG near cell I2 was reported to evoke monosynaptic EPSPs in simultaneously recorded respiratory motoneurons on both sides (Gariépy et al., 2012a). Also, we were unable to record any activity related to respiration around cell I1, despite being very careful in probing the area all around the giant cell. In the past, attempts at recording mesencephalic locomotor region neurons near cell I1 intracellularly showed no respiratory rhythmic activity (Ryczko et al., 2013, 2017).

Other evidence supports the location of the generator for the fast respiratory rhythm near cell I2. For example, in our experiments, only the injections of the sodium channel blocker, Xylocaine (2% lidocaine), aiming at cell I2 could abolish respiration, whereas the ones aiming at the dorsal level of cell I1 in the same animals had minimal effect on the respiratory rhythm frequency. Cinelli et al. (2013) obtained the exact opposite results, and we can only speculate on the reasons why. Through multiple attempts, we were unable to replicate those results, and even bilateral injections of Xylocaine over cell I1, or removing the whole area with lesions, could not abolish the fast respiratory rhythm recorded from vagal motoneurons. We should also add that unilateral injections of Xylocaine over cell I2 had some effect on respiration but only the addition of a second contralateral injection could completely abolish the rhythm within seconds. Similar results were previously obtained with Xylocaine injections and it was argued at the time that projections from the contralateral pTRG were not completely blocked by the unilateral injection in one pTRG, supporting the idea of a strong bilateral redundancy in the respiratory system of the lamprey (Gariépy et al., 2012a).

Further evidence in support of the localization of the pTRG around cell I2 comes from the fact that injections of tracers in the respiratory motoneurons that included at least the proximal portion of their huge distal dendritic tree retrogradely labeled populations of neurons in the rostral hindbrain, particularly in the region of the physiologically identified pTRG near cell I2 (Gariépy et al., 2012a). We further show here that injecting only the superficial layer containing the motoneuron cell bodies gave comparable results. Finding neurons that project to respiratory motoneurons in the area close to cell I2 is obviously of primary importance if the pTRG is to be located in that area. Also, one of the reasons it is important, in our view, to include the distal dendrites of the respiratory motoneurons in the tracer injections is that it was shown that intracellularly-labeled pTRG cells send axons to these dendrites deep in the ventro-lateral tegmentum of the caudal hindbrain (Gariépy et al., 2012a,b).

As we have now shown, the area dorsal to cell I1 is not necessary for the generation of the fast respiratory rhythm, but there are many indications that it is strongly connected to the respiratory networks. The best evidence of this is probably found in the abundant literature where the neurons located dorsal to cell I1 are shown to be implicated in many aspects of respiration (Gariépy et al., 2012b; Cinelli et al., 2013, 2014, 2016; 2017; 2020). We also must consider that in all our retrograde labeling experiments with injections in the respiratory motoneuron populations, cells located dorsal to cell I1 were strongly labeled on both sides of the brain, much like previously reported (Gariépy et al., 2012b; Cinelli et al., 2013). This strongly suggests that the neurons dorsal to cell I1 connect directly with the respiratory motoneurons and can thus affect their activity directly. We also have indications in our material that neurons dorsal to cell I1 could project to the area located close to cell I2. This comes from the experiments in which the location of the respiratory-related region around cell I2, as confirmed electrophysiologically, was injected with a marker that retrogradely labeled neurons dorsal to cell I1. Some evidence of this projection was also provided in a previous study where one of the cells dorsal to cell I1 was filled intracellularly and one of its axonal branches was followed to the pTRG near cell I2 (Gariépy et al., 2012b).

Our view here is that the neurons dorsal to cell I1 are part of the MLR (Brocard and Dubuc, 2003; Le Ray et al., 2003; Brocard et al., 2010; Smetana et al., 2010; reviewed in Ryczko and Dubuc, 2013) and probably serve as the neuronal substrate for the reported effects of MLR stimulation and associated locomotor activation on the respiratory generator (Gariépy et al., 2012b). Indeed, in the latter study by Gariépy et al. (2012b) we have intracellularly injected single neurons in the MLR. These experiments revealed strikingly complex projections from these neurons that reached the pTRG and respiratory motoneurons (facial, glossopharyngeal and vagal) on both sides. Such projections could clearly be responsible for any drug effects on respiration by injections into the MLR (cell I1 area). In addition, a recent study showed connections from the MLR to the respiratory CPG in mammals, indicating that such projections are not unique to lampreys (Hérent et al., 2023). Neurons in the area dorsal to cell I1 have been shown to express a variety of neurotransmitters, namely glutamate, D-serine, GABA, glycine, substance P, neuropeptide FF, and acetylcholine, many of which show effects on respiration (Rovainen, 1983; Pombal et al., 2001, 2006; Auclair et al., 2004; Robertson et al., 2007; Villar-Cerviño et al., 2008, 2013; Mutolo et al., 2010, 2011; Gariépy et al., 2012b; Cinelli et al., 2013, 2020).

One last set of data presented here in favor of the localization of the fast respiratory rhythm generator near cell I2 in the rostro-lateral hindbrain comes from lesion experiments. When the medial portion of the rostral hindbrain comprising the whole area around cell I1 and regions slightly more caudal to it were completely removed, the fast respiratory rhythm slowed in frequency but persisted.

Taken together these results eliminate the possibility, as proposed by Cinelli et al. (2013), that the region dorsal to cell I1 (MLR region) is the site where the fast respiratory rhythm is generated. It is noteworthy that the respiratory activity was not fully abolished after the removal of the rostral hindbrain as shown in the past (Kawasaki, 1979; Thompson, 1985; Russell, 1986). This suggests that some generator neurons are probably located even more caudally in the tegmentum than the area around cell I2. A distributed population of respiratory rhythm-generating neurons in the rostro-lateral hindbrain of lampreys has been suggested in the past, mostly based on lesion experiments (Kawasaki, 1979; Rovainen, 1985; Thompson, 1985). Our results here and those of others (Martel et al., 2007; Gariépy et al., 2012a) are in accord with a distributed fast rhythm generator, but with a larger role played by the most rostro-lateral portion of the distribution. In any case, further experiments are needed to record from individual rhythmic neurons in the area and to determine whether they have rhythmogenic properties (pacemaker or network).

### 4.2 On the slow rhythm generator in the caudal hindbrain

Because the spontaneous slow rhythm is synchronized on each side and it momentarily stops the fast rhythm (Martel et al., 2007), it is likely that the slow rhythm generator comprises neurons with local commissural and ascending projections. This was the premise for the anatomical experiments conducted in the present study. We indeed found neurons that project to the lateral tegmentum on the other side, others that project to the pTRG in the rostro-lateral hindbrain, and others that project to both regions. In mammals, respiration relies on Dbx1-derived glutamatergic interneurons in the pre-Bötzinger complex that have commissural axons crucial for bilateral synchronization (Bouvier et al., 2010). Moreover, the pre-Bötzinger complex sends ascending, descending, ipsi- and contralateral projections to other respiratory centers in the hindbrain (Tan et al., 2010). Other respiratory regions also have crossing, as well as ascending and descending projections (Bongianni et al., 1990; Janczewski and Karczewski, 1990; Goshgarian et al., 1991; Ezure et al., 2003; Duffin and Li, 2006; Tarras-Wahlberg and Rekling, 2009; Guyenet and Mulkey, 2010; Smith et al., 2013; Richter and Smith, 2014).

In the present study, we examined whether the neurons anatomically defined in the lateral tegmentum of the caudal hindbrain were involved in generating the slow rhythm by activating and inactivating them. We are now showing that activating the rostral, the middle, and the caudal parts of the caudal hindbrain lateral tegmentum triggered bursts that were characteristic of the slow rhythm. These results clearly demonstrate that the neurons involved in generating the slow rhythm are distributed along the rostro-caudal extent of the lateral tegmentum in the caudal half of the hindbrain. However, the higher stimulation intensity needed for eliciting a slow rhythm burst from the rostral region suggests that there may be a lower density of generator neurons in that region or that the neurons may be less excitable.

Our data also show that inactivation of any of the three sites temporarily abolishes the slow rhythm. This is particularly interesting because it shows that it is possible to stop the entire slow rhythm by inactivating only a small portion of the lateral tegmentum. When we conducted the same experiments in the whole brainstem, the slow rhythm was similarly abolished but not the fast rhythm. We found no subthreshold oscillations associated with the slow rhythm in respiratory motoneurons recorded intracellularly. These results suggest that the activating effects are not exerted on the motoneurons, but on premotor and/or generator neurons. There may be a need for enough generator neurons to be active to produce the slow rhythm (Hayes et al., 2012; Wang et al., 2014). In addition, the slow rhythm generator neurons may also provide recurrent excitation to each other, resulting in an increased excitability. A reduced excitation in a small group of generator neurons could then be sufficient to abolish the slow rhythm altogether. A similar mechanism, termed the “group pacemaker hypothesis” was proposed to explain the mechanisms underlying respiratory rhythm generation in mammals (Rekling and Feldman, 1998; Del Negro and Hayes, 2008; Del Negro et al., 2018).

Altogether, the present physiological experiments suggest that the neurons that were labeled by the anatomical tracers in the caudal hindbrain play a role in generating the slow respiratory rhythm in lampreys. To confirm this the activity and membrane properties of the neurons need to be characterized.

### 4.3 Comparison with other vertebrates

#### 4.3.1 Neurotransmitters involved

The fast rhythm in lampreys (Bongianni et al., 1999; Martel et al., 2007), the lung rhythm in amphibians (Chen and Hedrick, 2008), the inspiratory rhythm in the pre-Bötzinger complex (Greer et al., 1991; Bouvier et al., 2010), and the post-inspiratory rhythm in the post-inspiratory complex (PiCo) (Anderson et al., 2016) all appear to be dependent on glutamatergic transmission. The Phox2b-expressing pFRG/Pre-I neurons that play a role in active expiration are partly composed of glutamatergic neurons also expressing substance P/neurokinin-1 receptors (Onimaru et al., 2009). We have shown here that bath-application of glutamate antagonists stops the slow rhythm in the caudal hindbrain preparation, indicating that glutamatergic synaptic transmission plays a critical role in its generation. Interestingly, glutamatergic interneurons located along the facial, glossopharyngeal, and vagal motoneuronal pools were recently reported in lampreys (Cinelli et al., 2016). Their glutamatergic phenotype and their rostro-caudal distribution are remarkably similar to what we find here, and it suggests that these neurons could be involved in generating the slow rhythm, but further investigation is needed.

Neuropeptides are known to modulate the activity of neurons taking part in the generation of respiration (Bonham, 1995). In mammals, substance P depolarizes pre-inspiratory neurons of the retrotrapezoid nucleus/parafacial respiratory group (Onimaru et al., 2012) and increases the respiratory rhythm when injected in the pre-Bötzinger complex of rodents (Gray et al., 1999). The same effects are observed with regards to the lung rhythm in amphibians (Chen and Hedrick, 2008) and micro-injection of substance P in the pTRG of lampreys increases the fast respiratory rhythm (Mutolo et al., 2010). We now show that bath-application or micro-injections of substance P in the lateral tegmentum accelerate the slow rhythm in the caudal hindbrain preparation. In support of these effects, immunohistochemical experiments performed in the adult lamprey, *Petromyzon marinus*, showed that the region neighboring the respiratory motoneurons, where we found numerous slow rhythm generator candidate neurons, is rich in tachykinin-positive fibers (Auclair et al., 2004). In the latter study, the specific neurons of origin of the tachykininergic fibers were not identified, but many tachykinin-positive neurons are found throughout the brain, including the hindbrain. Some of the neurons were located in the dorsal column nuclei, the nucleus of the solitary tract, the octavo-lateral area, the rostral X motor nucleus, the medial reticular formation, the area lateral to the trigeminal motor nucleus near the sulcus limitans in the rostro-lateral hindbrain, and numerous cells in the isthmus, most located ventral to cell I1 and a few dorsal to it.

Local injections of DAMGO in the lateral tegmentum abolished the slow respiratory rhythm of lampreys in the caudal hindbrain preparation. These results concur with the general observation that μ-opioid agonists induce episodes of apnea when injected in the pre-Bötzinger complex of neonatal rats (Gray et al., 1999), in the lung oscillator of frogs (Vasilakos et al., 2005), or the pTRG of lampreys (Mutolo et al., 2007). No effects were observed when these agonists were injected in the site responsible for active expiration in mammals (retrotrapezoid nucleus/parafacial respiratory group) (Janczewski et al., 2002), or the buccal oscillator in frogs (Vasilakos et al., 2005). Based on its combined general response to glutamate, opioids, and substance P, the slow rhythm generator in lampreys appears to share comparable properties with the pre-Bötzinger complex of mammals and the lung oscillator of amphibians.

#### 4.3.2 Functional comparisons

The pTRG embryonic origin in the rostral hindbrain corresponds to rhombomeres 2 and 3 (pons) (Murakami et al., 2004; Gariépy et al., 2012a; Baghdadwala and Wilson, 2014; Funk, 2017; Milsom, 2018). Because of this, the pTRG would be more comparable to the pontine respiratory group of mammals, including the dorsolateral Kölliker-Fuse nucleus and the adjacent parabrachial nucleus (KF/PB) (Dutschmann and Dick, 2012). Even though the KF/PB nuclei contain respiratory rhythmic neurons (Ezure and Tanaka, 2006), they appear to be shaping and adapting the breathing pattern rather than generating respiration (Cohen, 1971; von Euler, 1977; St John and Zhou, 1991; Alheid et al., 2004; Rybak et al., 2008; Arata et al., 2010). The KF/PB also receive inputs from sensory related regions (Dutschmann and Dick, 2012), while a portion of their descending projections directly targets respiratory motoneurons (Fulwiler and Saper, 1984; Rikard-Bell et al., 1984, 1985). Both lamprey pTRG neurons and mammalian KF neurons are also sensitive to opioids (Levitt et al., 2015). For instance, activation of μ-opioid receptors in the KF region eliminates the post-inspiratory phase of the respiratory cycle and diminishes its frequency as well as the lungs tidal volume (Levitt et al., 2015). Lesions of the pontine respiratory group are also known to greatly disrupt respiration in mammals (Lumsden, 1923a,b; Bertrand et al., 1971; Feldman and Gautier, 1976; St-John and Paton, 2004).

The fast rhythm generated by the pTRG has traditionally been associated with mammalian respiration generated by the pre-Bötzinger complex (Martel et al., 2007; Mutolo et al., 2007; Missaghi et al., 2016), but it is also similar to the frog buccal rhythm, which also has a frequency of approximately 1 Hz. On the other hand, the lamprey slow rhythm has a frequency closer to that of the lung rhythm in frogs (approximately one event every minute) (Figure 1 in Wilson et al., 2002; Martel et al., 2007; Baghdadwala et al., 2015). Another respiratory rhythm in the mouse embryo, described as a slow inspiratory rhythm with augmented breaths (sighs), also shares similarities with the lamprey slow and the frog lung rhythms. Sighs show a period of approximately 1 min (1.3 ± 0.1 burst/min). They are generated by a distinct population of inspiratory neurons in the pre-Bötzinger complex and are characterized by a short post-sigh pause before the return of the eupneic bursts (Toporikova et al., 2015). Interestingly, a similar pause follows a slow rhythm event in lampreys (see Figure 3 in Martel et al., 2007).

All the data reported here strongly suggest that the slow rhythm generator neurons of lampreys are distributed along the rostro-caudal extent of the lateral tegmentum in the caudal hindbrain, spanning the equivalent of embryonic rhombomeres 4 to 7/8 (Murakami et al., 2004). From an embryonic standpoint, nuclei implicated in respiratory generation in mammals, like the retrotrapezoid nucleus/parafacial respiratory group and Bötzinger complex, arise from rhombomeres 4 and 5, whereas the pre-Bötzinger complex arises from rhombomere 7. In frogs, the data suggest that the lung oscillator arises from rhombomere 5 and the buccal oscillator from rhombomeres 7/8 (Baghdadwala et al., 2015). Moreover, the two oscillators are interdependent and could be viewed as a dual-oscillator network for respiration, spanning a large longitudinal area of the caudal hindbrain. Similarly, the respiratory centers in the caudal hindbrain of mammals (retrotrapezoid nucleus/parafacial respiratory group, Bötzinger complex, pre-Bötzinger complex, rostral ventral respiratory group, caudal ventral respiratory group) are described as a continuous succession of structures globally known as the ventral respiratory column (Feldman and Del Negro, 2006; Alheid and McCrimmon, 2008; Smith et al., 2009).

**FIGURE 15:**
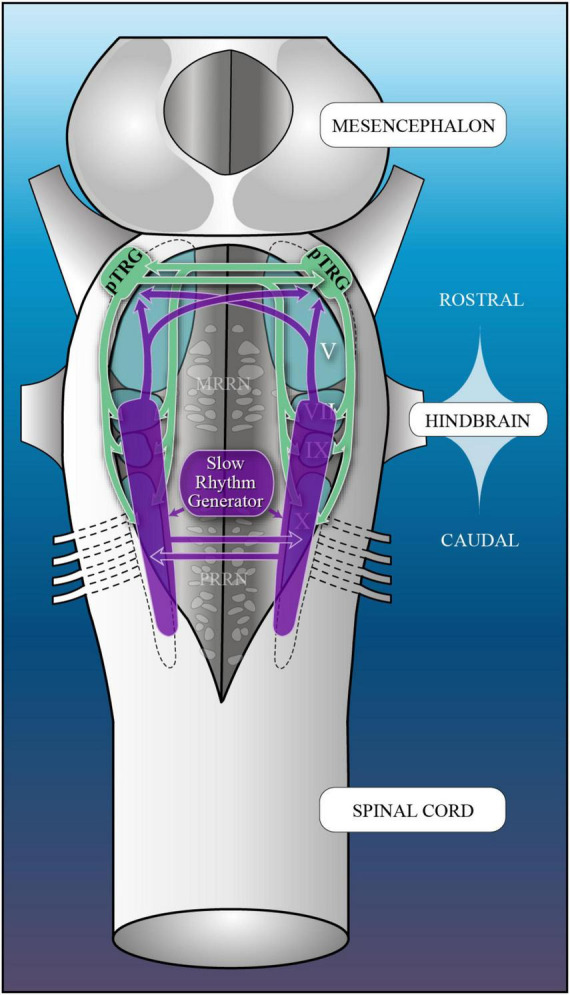
Proposed neural networks underlying the fast and slow respiratory rhythms in lampreys. Schematic illustration of the adult lamprey brainstem depicting the location of the respiratory rhythm generators. The neural networks and connectivity associated with the fast respiratory rhythm are illustrated in green. The neural networks associated with the slow respiratory rhythm are illustrated in purple. pTRG, paratrigeminal respiratory group; VII, facial motor nucleus; IX, glossopharyngeal motor nucleus; V, trigeminal motor nucleus; X, vagal motor nucleus; MRRN, middle rhombencephalic reticular nucleus; PRRN, posterior rhombencephalic reticular nucleus.

It was also shown that the slow rhythm generator of lampreys is sensitive to CO_2_/pH (Hoffman et al., 2016). According to the authors, the presence of central CO_2_/pH-sensitive chemoreceptors associated with the slow rhythm generator of lampreys may have provided an important substrate for the evolution of vertebrate air breathing. In mammals, CO_2_-sensitive neurons that express Phox2b are found in the retrotrapezoid nucleus/parafacial respiratory group, which is in the periphery of the VII motor nucleus in the rostral portion of the caudal hindbrain that corresponds to embryonic rhombomeres 4 and 5 (Onimaru and Homma, 2003; Onimaru et al., 2008, 2018). Others have demonstrated that CO_2_-sensitivity in the adult frog brainstem persists when the buccal oscillator is inhibited, but not the lung oscillator, located between the facial and glossopharyngeal nerves, in an area corresponding to embryonic rhombomere 5 (Leclère et al., 2012; Reed et al., 2018). Interestingly, it was shown that the fast rhythm in lampreys is sensitive to hypoxia (Rovainen, 1996), but not to CO_2_/pH (Hoffman et al., 2016). Although it has traditionally been compared to the generator of respiration in the pre-Bötzinger complex of mammals, the fast rhythm generator in lampreys is not sensitive to CO_2_ and originates from rhombomeres 2 and 3 in the rostral hindbrain. Consequently, the exact homology of the pTRG with respiratory centers in mammals remains to be fully established. In this context, it is possible that the slow rhythm generator in the caudal hindbrain of lampreys constitutes an unspecialized and distributed ancestral form of a respiratory generator that has evolved to become more complex, specialized, and multi-centered as vertebrates became air-breathing animals (Figure 15).

## 5 Conclusion

Although some of the neuronal mechanisms of respiratory pattern generation of the fast rhythm in lampreys are evolutionary conserved (Funk, 2017), the same can be said about the slow rhythm. It is possible that an unspecialized, distributed rhythm generating region located in the caudal hindbrain, such as the slow rhythm generator of lampreys, may have undergone substantial modifications through the course of evolution. It is less likely that the fast rhythm generator in the rostral hindbrain would have migrated more caudally to become the pre-Bötzinger complex (Hoffman et al., 2016; Milsom, 2018). We support the idea that the “mechanisms common and critical to vertebrate breathing arose through exaptation (Gould and Vrba, 1982) from those present in the basal vertebrate ancestor” (Hoffman et al., 2016, p. 8). Based on our current knowledge, we support the assertion that reorganizations and specializations of the slow rhythm generator of lampreys have occurred through evolution and that it could represent a precursor of air-breathing vertebrate neural networks located in the caudal hindbrain.

## Data availability statement

The raw data supporting the conclusions of this article will be made available by the authors, without undue reservation.

## Ethics statement

The animal study was approved by the Comité de Déontologie de l’Expérimentation sur les Animaux de l’Université de Montréal. The study was conducted in accordance with the local legislation and institutional requirements.

## Author contributions

RD: Conceptualization, Formal analysis, Funding acquisition, Supervision, Validation, Writing—original draft, Writing—review and editing, Methodology. KM: Conceptualization, Data curation, Formal analysis, Investigation, Methodology, Validation, Writing—original draft, Writing—review and editing. P-AB: Conceptualization, Data curation, Formal analysis, Investigation, Methodology, Validation, Writing—original draft, Writing—review and editing. J-PL: Conceptualization, Data curation, Formal analysis, Investigation, Methodology, Validation, Writing—original draft, Writing—review and editing. JM: Investigation, Methodology, Writing—review and editing. MG: Investigation, Methodology, Writing—review and editing. SC: Investigation, Methodology, Writing—review and editing. OM: Investigation, Methodology, Writing—review and editing. FA: Conceptualization, Formal analysis, Investigation, Methodology, Supervision, Visualization, Writing—original draft, Writing—review and editing.
